# Corynanthean-Epicatechin Flavoalkaloids from *Corynanthe pachyceras*

**DOI:** 10.3390/molecules25112654

**Published:** 2020-06-07

**Authors:** Tapé Kouamé, Aboua Timothée Okpekon, Nicaise F. Bony, Amon Diane N’Tamon, Jean-François Gallard, Somia Rharrabti, Karine Leblanc, Elisabeth Mouray, Philippe Grellier, Pierre Champy, Mehdi A. Beniddir, Pierre Le Pogam

**Affiliations:** 1 Université Paris-Saclay, CNRS, BioCIS, 92290 Châtenay-Malabry, France; ulrikkouam@gmail.com (T.K.); tamondianemarina@yahoo.fr (A.D.N.); somia.rharrabti@universite-paris-saclay.fr (S.R.); karine.leblanc@universite-paris-saclay.fr (K.L.); pierre.champy@universite-paris-saclay.fr (P.C.); 2 Laboratoire de Chimie Organique et de Substances Naturelles (LCOSN), UFR Sciences des Structures de la Matière et Technologie, Univ. FHB, 22 BP 582 Abidjan 22, Côte d’Ivoire, France; okpekon@yahoo.fr; 3 Département de Chimie Analytique, Minérale et Générale, Technologie Alimentaire, UFR Sciences Pharmaceutiques et Biologiques, Univ. FHB, 06 B. P. 2256 Abidjan 06, Côte d’Ivoire, France; bonynicaise@yahoo.fr; 4 Institut de Chimie des Substances Naturelles, CNRS, ICSN UPR 2301, Université Paris-Saclay, 21 Avenue de la Terrasse, 91198 Gif-sur-Yvette, France; jean-francois.gallard@cnrs.fr; 5 Muséum National d′Histoire Naturelle, Unité Molécules de Communication et Adaptation des Micro-organismes, UMR7245, CP54, 57 Rue Cuvier, 75005 Paris, France; elisabeth.mouray@mnhn.fr (E.M.); philippe.grellier@mnhn.fr (P.G.)

**Keywords:** rubiaceae, *Corynanthe pachyceras*, flavoalkaloids, monoterpene indole alkaloids, molecular networking

## Abstract

Epicatechocorynantheines A and B, and epicatechocorynantheidine were isolated from the stem bark of *Corynanthe pachyceras*. These molecules were pinpointed, and their isolation streamlined, by a molecular networking strategy. The structural elucidation was unambiguously accomplished from HRMS and 1D/2D NMR data. These compounds represent the first examples of corynanthean-type alkaloids tethered with a flavonoid. Epicatechocorynantheidine notably instigated two connections between the monoterpene indole alkaloid and the flavonoid, yielding an unprecedented octacyclic appendage. These flavoalkaloids exerted moderate antiplasmodial activities.

## 1. Introduction

**Figure 1:**
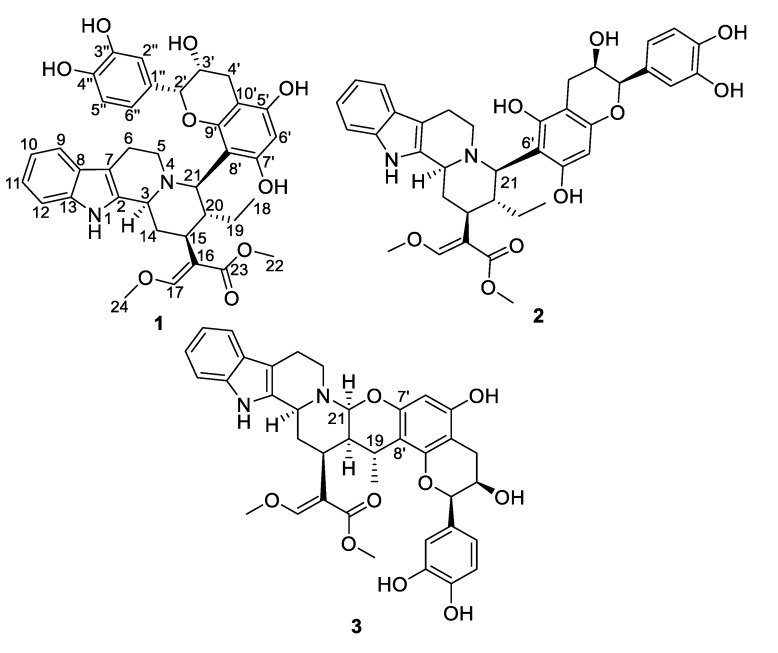
Chemical structures of compounds **1**–**3**.

As one of the five most species-rich flowering plant families, Rubiaceae plants are sustained by more than 620 genera accounting for 13,000 species, which are essentially distributed in tropical and subtropical areas [1]. Plants in this family are endowed with various and significant bioactivities that paved the way for extensive campaigns of phytochemical investigations within this privileged biota [2]. These thorough studies have revealed a variety of indolomonoterpenic alkaloids appendages, anthraquinones, and triterpenes to occur in this group, with some iconic compounds that are widely used in clinical practice such as the antimalarial quinine from *Cinchona ledgeriana* and the antihypertensive drug rhynchophylline from *Uncaria rhyncophylla* [3]. From a chemical viewpoint, *Corynanthe pachyceras* K. Schum represents an untapped member of this fascinating family despite its being used in traditional medicine as intoxicant, local anesthetic and febrifuge [4]. Accordingly, the sole report that has dealt with this plant so far reported the occurrence of yohimbine and corynantheine-type indolomonoterpenic alkaloids [5]. In the frame of our stern efforts directed towards the discovery of new indolomonoterpenic alkaloids [6,7,8], and seizing the opportunity of the recent GNPS [9] upload of the MIADB repository [10], a molecular-networking-based dereplication strategy was undertaken on the bark of *Corynanthe pachyceras*. This publication reports the tandem mass-spectrometric-streamlined isolation of epicatechocorynantheines A and B (**1** and **2**), and of epicatechocorynantheidine (**3**) (Figure 1), representing the first examples of corynanthean-type alkaloids tethered with a flavonoid core and the second natural occurrence of an indolomonoterpenic alkaloid/flavonoid hybrid, following the seminal report of uncariagambiriines A−C [11,12] from the Rubiaceae *Uncaria gambir*. The octacyclic scaffold of epicatechocorynantheidine (**3**) is related to a so-far unique double connection between the building blocks of a monoterpene indole alkaloid-flavonoid hybrid.

## 2. Results and Discussion

### 2.1. Molecular Networking-Based Prioritization of the Isolation Workflow

To reach a comprehensive insight into the indolomonoterpenic alkaloid content of *C. pachyceras*, the alkaloid and crude ethanolic extracts of its stem bark were profiled by HPLC-HRMS². These data were subsequently preprocessed and organized following the feature-based molecular networking workflow [13]. Detecting the sought-after compounds in both alkaloidic and ethanolic extracts demonstrated their genuine origin ruling out the possibility of artifacts being formed throughout alkaloid fractionation. The dereplication of these extracts against the MIADB-implemented GNPS overall revealed five hits, related to either yohimban or corynanthean series, corresponding to formerly reported compounds of *C. pachyceras* or diastereoisomers thereof, which are not easily distinguishable based on tandem mass-spectrometric analyses. A cautious examination of the generated molecular network revealed the presence of a molecular family constituted of four nodes with relatively high molecular masses that revealed no putative hit against GNPS spectral libraries, with three of them appearing amenable to isolation in sufficient amount for full structure elucidation: two features at *m/z* 657.2888 and 657.2896 (**1**–**2**), which were only distinguished following MZmine 2 pre-processing [14], and a further one at *m/z* 655.2726 (**3**). Depending on the type of extract, these compounds either formed a cluster on their own or loosely attached to an important constellation containing yohimban and corynanthean alkaloids (Figures S1 and S2, Supporting Information). The lack of diagnostic isotopic pattern for doubly charged ion species in their mass spectrum, as encountered in most dimeric indolomonoterpenic alkaloid, strengthened our interest in isolating these species owing to their elevated molecular masses. Within the tolerance settings of the instrument (i.e., 5 ppm *m/z* tolerance and lowest i-fit below 2), [15] only one molecular formula could be proposed for these two isomeric pairs (*viz.* C_37_H_40_N_2_O_9_) for **1** and **2** and C_37_H_38_N_2_O_9_ for **3**). Gratifyingly, their search against the *Dictionary of Natural Products* [16] returned no hit likely to occur in Rubiaceous plants, providing a strong rationale for their mass-spectrometric-streamlined isolation. 

### 2.2. Structure Elucidation

**Figure 2:**
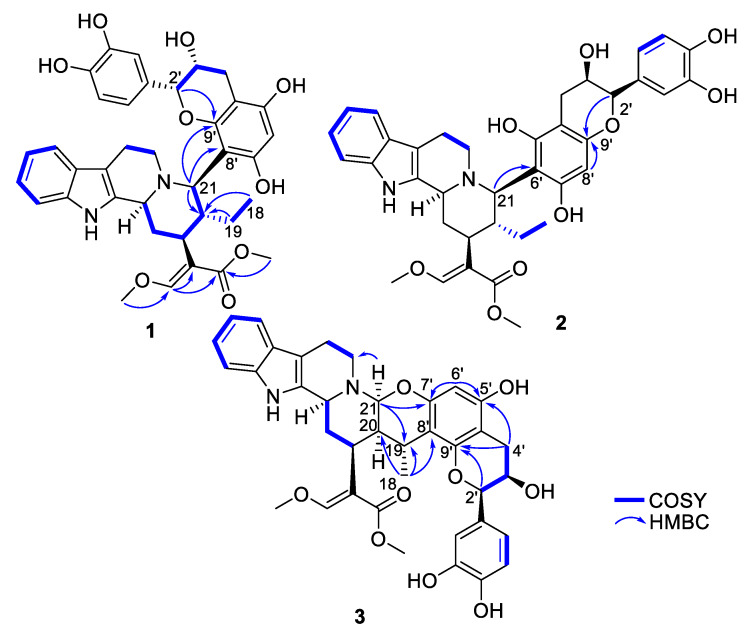
Key COSY and HMBC correlations of compounds **1**–**3**.

Epicatechocorynantheine A (**1**) was isolated as a grey amorphous solid (Figure 1). The molecular formula of **1** was assigned as C_37_H_40_N_2_O_9_ by the HRESIMS ion (*m/z* 657.2811, [M + H]^+^, calcd 657.2807), for 19 indices of hydrogen deficiency, in conjunction with the ^13^C NMR spectrum. The ^1^H NMR data revealed the diagnostic signals of an olefinic proton (δ_H_, 7.58, 1H, s, H-17), an *ortho*-disubstituted phenyl system (δ_H_ 7.22, 1H, d, *J* = 7.0 Hz, H-9; δ_H_ 7.31, 1H, d, *J* = 7.0 Hz, H-12; δ_H_ 7.11, 1H, t, *J* = 7.0 Hz, H-11; δ_H_ 6.94, 1H, t, *J* = 6.9 Hz, H-10), two methoxy groups at δ_H_ 3.66 (3H, s, OCH_3_-22) and at δ_H_ 3.98 (3H, s, OCH_3_-24), and an ethyl group (δ_H_ 1.24 and 1.34, 2H, m, H-19; δ_H_ 0.75, 3H, t, *J* = 7.5 Hz, H-18). The ^13^C NMR spectrum, jointly with the HSQC spectrum, revealed carbons allocated to three methyls (two being oxygen-bonded), five methylenes, one oxygenated olefinic carbon, six aliphatic methines, eight aromatic methines, seven tertiary sp² carbons (including five oxygenated carbons), six aromatic/olefinic quaternary carbons, and an ester carbonyl carbon. The COSY spectrum led to the assignment of two spin systems, i.e., –CH-CH_2_-CH-CH(CH_2_-CH_3_)-CH and –CH_2_-CH_2_ (Figure 2). These spectroscopic features, along with HMBC correlations that also established the presence of a *β*-methoxyacrylate moiety, suggested **1** to be a tetracyclic corynanthean alkaloid [5], except that the diastereotopic methylene at C-21 was replaced by a methine, hinting that the rest of the molecule should be anchored at this specific position. Corynanthean-type indolomonoterpenic alkaloids containing a non-rearranged monoterpene unit retain the *α*-configuration for H-15 [17,18]. As the structurally related yohimbanes [19], corynantheine-type alkaloids are divided into four different relative configurations: normal (3*α*, 15*α*, 20*β*), pseudo (3*β*, 15*α*, 20*β*), allo (3*α*, 15*α*, 20*α*), and epiallo (3*β*, 15*α*, 20*α*) [20]. The relative configuration of **1** could be assigned based on the analysis of ROE correlations and coupling constant values interpretation. The ROE crosspeaks (S12, Supporting Information) between the aliphatic methine proton resonating at δ_H_ 3.13 (H-15) to the methine at δ_H_ 3.90 (H-3) and to both the diastereotopic methylene signals at δ_H_ 1.24/1.34 (H_2_-19) and to the methyl group at δ_H_ 0.75 (H_3_-18) determined the pertaining of **1** to the normal series, consistent with the coupling constant values of H-3 and H-15 [19]. This stereochemical arrangement is in line with most indolomonoterpenic alkaloids formerly reported to occur in *C. pachyceras* [5]. At last, the methine proton resonating at δ_H_ 4.55 (H-21) could be determined as *α*-oriented based on its ROE crosspeaks with both H-3 and H-15, while its elevated vicinal coupling constant value validated its *α*-axial orientation. The remaining part of the molecule stood for C_15_H_13_O_6_ and comprised nine further indices of hydrogen deficiency. The ^1^H NMR spectrum, along with COSY correlations, exhibited two additional spin systems: an ABX spin system consisting of three aromatic protons at H-2′′ (δ_H_ 7.05), H-5′′ (δ_H_ 6.79), and H-6′′ (δ_H_ 6.87) disclosing *meta*, *ortho* and *ortho*-*meta* coupling patterns; and an ABMX spin system comprising a diastereotopic pair of methylenic protons at δ_H_ 2.82 (1H, d, *J* = 16.8 Hz, H-4′a) and 2.94 (1H, s, dd, *J* = 16.8, 4.5 Hz, H-4′b), an oxygenated methine at δ_H_ 4.20 (1H, t, *J* = 4.5 Hz, H-3′), and a further oxygenated methine at δ_H_ 4.91 (1H, d, *J* = 4.5 Hz, H-2′), supporting the presence of a typical flavanol of the epicatechin type, as further validated by the HMBC correlations from H-2′ to C-1′′ (δ_C_ 131.9), C-2′′ (δ_C_ 116.0), and C-6′′ (δ_C_ 120.1). The 2′,3′-*cis* configuration could be mitigated based on the low magnitude of their vicinal coupling constant value [21], and also on the key H-2′/H-3′ ROE crosspeak. The pentasubstituted A-ring contained the two expected hydroxyl substituents along with an aromatic methine resonating at δ_H_ 6.11 (δ_C_ 96.3, H-6′), a ^13^C NMR chemical shift diagnostic of the presence of two *ortho*-oxygen functionalities [22]. Likewise, the quaternary carbon resonating at δ_C_ 100.6 (C-8′) indicated it being flanked by two oxygenated moieties [23], in line with flavonoid oxygenation pattern. The HMBC correlations from both the oxymethine proton at δ_H_ 4.91 (H-2′) and the methine proton at δ_H_ 4.55 (H-21) to C-9′ (δ_C_ 156.3) established C-21/C-8′ connectivity (Figure 2). Epicatechocorynantheine A (**1**) was established as indicated in Figure 1.

Compound **2** was obtained as a white amorphous solid with a molecular formula of C_37_H_40_N_2_O_9_, established by the HRESIMS ion at *m/z* 657.2813 [M + H]^+^, indicating **2** is an isomer of **1**. Both its ^1^H and ^13^C NMR spectroscopic data were similar to those of **1**, instantly revealing identical corynantheidine and epicatechin building blocks. Only some slight NMR shifts focused on epicatechin A-cycle could be observed (Table 1), hinting that **2** might differ from **1** by connecting C-21 to C-6′ as an alternative nucleophilic site. This assumption was ascertained based on the long-range heteronuclear correlation from both the aromatic proton at δ_H_ 6.07 (H-8′) and the oxymethine proton at δ_H_ 4.84 (H-2′) to C-9′ (δ_C_ 156.1). These spectroscopic features defined **2**, namely epicatechocorynantheine B, as shown in Figure 1.

Compound **3**, isolated as a yellow amorphous solid, gave a molecular formula of C_37_H_38_N_2_O_9_ based on its positive-ion mode HRESIMS data, which showed a [M + H]^+^ ion peak at *m/z* 655.2654, differing from **1** by two mass units. Examination of the ^1^H and ^13^C NMR spectra of **3** revealed signal patterns similar to those of **1**, straightforwardly determining a corynanthean and an epicatechin subunits. A deeper analysis of the NMR data of **3** revealed two salient differences with those of **1**. The ^13^C NMR chemical shift of C-21 was 22 ppm downfield shifted compared to that of **1** so that the chemical shifts of this methine (δ_H_ 4.31, δ_C_ 89.2) were diagnostic of it being constrained between a nitrogen and an oxygen atom [24]. These spectroscopic features hinted that C-21 should be connected to an oxygen atom of the epicatechin building block, as further backed up by the HMBC correlation from the methine at δ_H_ 4.31 to an oxygenated *sp*^2^ carbon resonating at δ_C_ 152.4, which might either be C-5′ or C-7′. The ^1^H NMR data also revealed that H-19 diastereotopic methylene signals were replaced by a methine resonating at δ_H_ 3.04/δ_C_ 27.2 which, along with molecular formula and double bond equivalent requirements, suggested that the corynanthean and epicatechin subunits were bonded *via* a second interunit connectivity instigated from this position to create a cycle that would account for the last missing index of hydrogen deficiency. This assumption was validated based on the long-range heteronuclear correlation from this methine proton signal at δ_H_ 3.04 to an aromatic carbon resonating at δ_C_ 109.2, which was diagnostic of the presence of two *ortho*-oxygen functionalities (either C-6′ or C-8′). Three different planar structures remained compatible with these NMR landmarks: establishing a C-19–C-6′ bond leaves the possibility to instigate a connectivity with either of the *ortho* phenolic functions located at C-5′ or C-7′ introducing two possible regioisomers, and a third and last planar candidate could be reached by connecting C-19 to C-8′ which could only result in ring closure following a C-21–HO–C-7′ bonding due to this position being the sole *ortho* phenolic group (S38, Supporting Information). The HMBC crosspeaks between epicatechin A-cycle aromatic proton resonating at δ_H_ 6.03 to the oxygenated sp² carbons resonating at δ_C_ 156.0 (C-5′) and 152.4 (C-7′), along with the joint correlations of the oxymethine proton H-2′ and of the diastereotopic methylenic signals at δ_H_ 2.73/2.90 (H_2_-4′) to the third A-cycle epicatechin oxygenated aromatic carbon at δ_C_ 153.7 (C-9′), located the aromatic proton at C-6′, determining the planar structure of **3** as shown in Figure 1. The relative configurations of both the monoterpene indole alkaloid and epicatechin components were assigned based on the analysis of vicinal coupling constant magnitudes and subsequent interpretation of ROESY correlations (S37, Supporting Information). The ROESY crosspeaks observed between H-3, H-15, H-21, and H_3_-18 defined their alpha-orientation. Taking into account the *α*-axial orientation of H-21, and its broad singlet status determined the *α*–equatorial orientation of H-20, a deduction also supported by the H-20/H_3_-18 ROE crosspeak, defining the indolomonoterpenic alkaloid component of **3** as corynantheidine (allo series). Thus, the structure of **3**, namely epicatechocorynantheidine, was elucidated as shown in Figure 1.

Epicatechocorynantheines A and B and epicatechocorynantheidine exerted moderate antiplasmodial activity against the chloroquine-resistant strain FcB1 of *P. falciparum* with respective IC_50_ values of 23.0 ± 0.0; 10.5 ± 3.5; and 22.0 ± 2.0 μM.

### 2.3. Biosynthetic Pathway Hypothesis

Biosynthetic schemes to reach **1**–**3** are proposed in S39, Supporting Information. Compounds **1** and **2** could be obtained following a nucleophilic attack instigated by either epicatechin C-8′ or C-6′, respectively, to the C-21 iminium ion derived from corynantheidine. As per **3**, 4,21-dehydrogeissoschizine methylether as a precursor would enable a 1,4-Michael addition initiated from C-8′ to the olefinic carbon C-19, which would result in installing a Δ^20,21^ moiety. Next, the addition of a proton to the nucleophilic enamine moiety at C-20 might yield an iminium ion, prone to undergo a second, intramolecular, nucleophilic attack, triggered by 7′-OH to afford the central dihydropyran nucleus of **3**. Closely related 4,21-dehydrocorynantheine alkaloids were proposed to step in the biosynthesis of ajmalicine alkaloids [25]. Likewise, 4,21-dehydrogeissoschizine is known from natural source, and it was shown that it could cyclize into cathenamine [26], following a mechanism analogous to that proposed to step in the biosynthesis of **3**. Regarding our specific example, the possibility to undertake this intramolecular 1,4 addition is rendered impossible by the methylation of the occurrence of a methoxy group at C-17, favoring instead the intermolecular scenario with epicatechin that paves the way to **3**. Notably, 4,21-dehydrogeissoschizine methylether was already reported from Rubiaceous source [27]. Epicatechin was already reported from *C. pachyceras* as well [28].

In stark contrast with the huge number of both alkaloids and flavonoids, flavoalkaloids constitute an intriguingly small phytochemical group with pyrrole-, pyrrolidine-, piperidine-, and piperidinone flavoalkaloids being the most frequent scaffolds. Accordingly, the small group of indole flavoalkaloids, which ushered in the late 1990s with the isolation of (−)-licorice glycoside E from *Glycyrrhiza uralensis* [29] was so far represented by only eight structures; two oxindole-flavoalkaloids from *Aesculus hippocastanum* [30,31] (one of which being only tentatively identified), (+)-lotthanongine from *Trigonostemon reidioides* [32], yuremamine isolated from *Mimosa tenuiflora* [33] along with the aforementioned uncariagambiriines A−C.

## 3. Materials and Methods

### 3.1. General Experimental Procedures

Optical rotations were obtained at 25 °C on a Polar 32 polarimeter. UV spectra were recorded at 25 °C on a Jasco J-810 spectropolarimeter. The NMR spectra were recorded on a Bruker AM-300 (300 MHz), AM-400 (400 MHz), and AM-600 (600 MHz) (Bruker, Karlsruhe, Germany) equipped with a microprobe TXI 1.7 mm. NMR spectrometers were calibrated using solvent residual signals as references. HMBC analyses were optimized for a coupling constant [*CNST13*] of 6 Hz (**2** in DMF-d_7_), 10 Hz (**1** and **3**), or 13 Hz (**2** in CD_3_OD). For NOESY of **1**, mixing time (*D8*) = 500 ms, for NOESY of **2** and **3**, mixing time (*D8*) = 300 ms. Analytical HPLC runs were carried out using an Agilent LC-MS system consisting of an Agilent 1260 Infinity HPLC hyphenated with an Agilent 6530 ESI-Q-TOF-MS operating in positive polarity. Silica 330 and 24 g Grace cartridges were used for flash chromatography using an Armen instrument spot liquid chromatography flash apparatus. Sunfire^®^ preparative C_18_ columns (150 × 4.6 mm, i. d. 5 μm, Waters) were used for preparative HPLC separations using a Waters Delta Prep (Waters Co., Milford, MA, USA) consisting of a binary pump (Waters 2525) and a UV-visible diode array detector (190–600 nm, Waters 2996). 

### 3.2. Plant Material

*Corynanthe pachyceras* K. Schum. whole plants were collected in the Agbo 2 area of Akoupe (GPS N 5.5017210, E −4.243631) in April 2018. The botanical identification was performed at the Centre National de Floristique (CNF)-Université Félix Houphouët Boigny–Abidjan (Côte d’Ivoire). A voucher specimen (No. OAT-CP-2018) is kept at the herbarium of CNF.

### 3.3. Chromatographic and Mass-Spectrometric Analysis

Samples were analyzed using an Agilent 6530 Accurate-Mass Q-TOF coupled with a 1260 Agilent Infinity LC system equipped with a Sunfire^®^ C_18_ column (150 × 2.1 mm ; i. d. 3.5 μm, Waters, Milford, MA, USA) with a flow rate of 0.25 mL/min. Full scan mass spectra were acquired in the positive-ion mode in a mass range of *m/z* 100 to 1200 Da, with the capillary temperature at 320 °C, source voltage at 3.5 kV, and a sheath gas flow rate at 10 L/min. Capillary, fragmentor, and skimmer voltages were set at 3500 V, 175 V, and 65 V respectively. Four scan events were used: positive MS, mass range encompassing *m/z* 100–1200, and three data-dependent MS/MS scans of the five most intense ions from the first scan event. Three collision energies (*viz.* 30, 50, and 70 eV) were used for MS/MS data generation. Purine (C_5_H_4_N_4_, *m/z* 121.050873 and HP-0921 (hexakis (*1 H*, *1 H*, *3 H*-tetrafluoropropoxy)-phosphazene C_18_H_18_F_24_N_3_O_6_P_3_, *m/z* 922.009798) were used as internal lock masses. Full scans were acquired at a resolution of 10,000 (*m/z* 922) and 4000 (*m/z* 121). A permanent MS/MS exclusion list criterion was established to prevent oversampling of the internal calibrant.

### 3.4. MZmine Data Pre-Processing and Molecular Networking Generation

The MS/MS data file were converted from the .d in-house Agilent data format to .mzXML thanks to the MS Convert Software, as included in ProteoWizard package [34]. All mzXML files were further submitted to the MZmine2v.52 workflow. The mass detection was performed with a mass detection threshold at 1.0E4. The ADAP chromatogram builder was obtained using a minimum group scan size of 4, a group intensity threshold of 1.0E4, a minimum highest intensity of 1.0E5 and *m/z* tolerance of 0.05 Da or 5 ppm [35]. The ADAP wavelets deconvolution algorithm was applied with the following settings: S/N threshold = 10, minimum feature height = 3, coefficient/area threshold = 3, peak duration range 0.02–4.0 min, RT wavelet range 0.02–0.6. MS/MS scans were paired using a *m/z* tolerance range of 0.02 Da and a RT tolerance range of 1.0 min. The chromatogram was deisotoped using the isotopic peak grouper algorithm with a *m/z* tolerance of 0.02 Da or 10 ppm and a RT tolerance of 1.0 min. The MGF preclustered data file and the corresponding .csv metadata file (for RT, areas, and formulas integration) were exported using the dedicated built-in options. The raw data files related to the LC-MS/MS analysis of the crude extract were deposited on the public MassIVE repository under the accession number MSV000085397. A molecular network was then created using the online workflow hosted at GNPS. The data were then clustered with MS-Cluster with a parent ion mass tolerance of 0.05 Da and a fragment ion mass tolerance of 0.05 Da to generate consensus spectra. Consensus spectra containing less than 1 spectrum were discarded. A network was then created where edges were filtered to have a minimal cosine score of 0.6 with at least six matched fragment peaks. Edges were further maintained if each of the nodes appeared in each other respective top 10 most similar nodes. When searching against GNPS libraries, matches between network spectra and library spectra required a cosine score of 0.6 and at least six matched peaks. The generated molecular network was visualized using Cytoscape 3.5.1 [36].

### 3.5. Extraction and Purification of Compounds

The dried barks of *C. pachyceras* (3.0 kg) were milled and extracted by maceration with EtOH (3 × 3 L, 24 h each, 40°C, atmospheric pressure). The EtOH extract was concentrated in vacuo at 40 °C to afford 200 gr of dry residue that was dissolved in 6 M NH_4_OH. This solution was submitted to liquid-liquid extraction with EtOAc (600 mL × 4). The EtOAc phase was extracted using a solution of sulfuric acid 1 M (300 mL × 3). This aqueous phase was alkalinized using 6M NH_4_OH and extracted with DCM to yield 32 g of an alkaloid extract. The dry residue was submitted to flash chromatography using a Silica 330 g Grace cartridge with a gradient of CH_2_Cl_2_/MeOH (1:0 to 0:1) at 130 mL/min to afford five fractions based on TLC profiles. Fraction F8 (2 g) was submitted to flash chromatography using a Silica 24 g Grace cartridge with the same gradient of CH_2_Cl_2_/MeOH (1:0 to 0:1) at 28 mL/min to afford five fractions based on TLC profiles. Fraction F8-6 (160 mg) was fractionated by preparative HPLC separation using a gradient of ACN-H_2_O with 0.1% FA (99:1 to 7:3 to afford **1** (26 mg). Fraction F8-7 (363 mg) was fractionated by preparative HPLC separation using the same gradient system of ACN-H_2_O with 0.1% FA (99:1 to 6:4) to afford **2** (17 mg), and **3** (15 mg).

*Epicathechocorynantheine A* (**1**). Grey amorphous solid; [*α*]_D_^20^ -140.6 (*c* 0.6, MeOH); UV (MeOH), *λ_max_* (log *ε*) 212 (3.6), 266 (4.5), 342 (3.8) nm; IR (*ν_max_*) 3422, 3186, 2843, 1457, 1095, 1051, 746 cm^−1^; ^1^H and ^13^C NMR data, see Table 1; HRESIMS *m/z* 657.2811 [M + H]^+^ (calcd for C_37_H_41_N_2_O_9_, 657.28066). MS/MS spectrum was deposited in the GNPS spectral libraries under the identifier: CCMSLIB00005721087.

*Epicathecocorynantheine B* (**2**). Brown amorphous solid; [*α*]_D_^20^ - 20.0 (*c* 0.1, MeOH); UV (MeOH), *λ_max_* (log *ε*) 232 (4.0), 290 (3.6) nm; IR (*νmax*) 3373, 2962, 2846, 1438, 1003, 744 cm^−1^; ^1^H and ^13^C NMR data, see Table 1; HRESIMS *m/z* 657.2813 [M + H]^+^ (calcd for C_37_H_41_N_2_O_9_, 657.28066). MS/MS spectrum was deposited in the GNPS spectral libraries under the identifier: CCMSLIB00005721088.

*Epicathecocorynantheidine* (**3**). Yellow amorphous solid; [*α*]_D_^20^ - 50.0 (*c* 0.1, MeOH); UV (MeOH), *λ_max_* (log *ε*) 228 (4.3), 262 (3.7), 310 (3.5) nm; IR (*νmax*) 3414, 3219, 2359, 1638, 1351, 1287, 774 cm^−1^; ^1^H and ^13^C NMR data, see Table 1; HRESIMS *m/z* 655.2654 [M + H]^+^ (calcd for C_37_H_39_N_2_O_9_, 655.26501 ). MS/MS spectrum was deposited in the GNPS spectral libraries under the identifier: CCMSLIB00005721089.

### 3.6. Antiplasmodial Activities

The chloroquine-resistant strain FcB1/Colombia of *Plasmodium falciparum* was obtained from the National Museum Natural History collection, Paris, France (n°MNHN-CEU-224- PfFCB1). Parasites were maintained in vitro in human erythrocytes in RPMI 1640 medium supplemented by 8% (*v*/*v*) heat-inactivated human serum at 37 °C under an atmosphere of 3% CO_2_, 6% O_2_, and 91% N_2_. Human red blood cells and serum were provided by the Etablissement Français du Sang Ile de France under the C-CPSL-UNT approval n°13/EFS/126. In vitro drug susceptibility was measured by [^3^H]-hypoxanthine incorporation as described previously.1 Stock solutions of drugs were prepared in DMSO. Compounds were serially diluted two-fold with 100 µL culture medium in 96-well plates. Asynchronous parasite cultures (100 µL, 1% parasitemia and 1% final hematocrit) were then added to each well and incubated for 24 h at 37 °C prior to the addition of 0.5 µCi of [^3^H]-hypoxanthine (GE Healthcare; 1 to 5 Ci mmol/mL) per well. After a further incubation of 24 h, plates were frozen and thawed. Cell lysates were then collected onto glass-fiber filters and counted in a liquid scintillation spectrometer. The growth inhibition for each compound concentration was determined by comparison of the radioactivity incorporated in the treated culture with that in the control culture maintained on the same plate. The concentration causing 50% growth inhibition (IC_50_) was obtained from the compound concentration-response curve and the results were expressed as mean values ± standard deviations as determined from three independent experiments. Chloroquine diphosphate, used as a positive control, was purchased from Sigma (Saint-Quentin Fallavier, France, purity > 99%).

## 4. Conclusions

A molecular networking strategy was implemented on *Corynanthe pachyceras* stem bark alkaloidic extract, streamlining the isolation of three chimeric structures pertaining to the rare phytochemical class of flavoalkaloids. These structures are unique in containing a corynanthean subunit as their monoterpene indole alkaloid component, accounting for the unprecedented carbon scaffold. Undoubtedly, the octacyclic appendage of epicatechocorynantheidine, resulting from the instigation of two distinct bonds between the monoterpene indole alkaloid and the epicatechin subunit, is the most original structure being described herein.

## Figures and Tables

**Table 1:** ^1^H and ^13^C NMR Spectroscopic Data for **1**–**3** in CD_3_OD.

	1*^a^*		2 *^b^*		3 *^a^*	
Position	δ_H,_ mult. (*J*, Hz)	δ_C_	δ_H,_ mult. (*J*, Hz)	δ_C_	δ_H,_ mult. (*J*, Hz)	δ_C_
2	-	131.2	-	132.0	-	135.1
3	3.90, bd (11.8)	63.8	3.92, m	62.2	3.75, d (13.0)	59.2
5	2.79, m 3.53, m	50.9	2.90, m 3.48, m	51.0	2.50, m 4.11, m	48.0
6	2.77, m 2.95, m	20.9	2.75, m 2.90, m	21.8	2.81, m 2.99, m	22.1
7	-	107.1	-	107.8	-	108.5
8	-	127.2	-	127.7	-	128.4
9	7.22, d (7.0)	119.0	7.40, d (7.0)	118.9	7.41, d (7.2)	118.7
10	6.94, t (7.0)	120.6	7.01, t (7.0)	120.2	6.97, t (7.2)	119.8
11	7.11, t (7.0)	123.1	7.10, t (7.0)	122.7	7.04, t (7.2)	122.0
12	7.31, d (7.0)	112.4	7.33, d (7.0)	112.3	7.28, d (8.0)	112.0
13	-	138.3	-	138.3	-	138.2
14a 14b	2.26, d (11.8) 2.47, td (11.8, 12.9)	32.3	2.26, d (12.1) 2.39, td (12.7, 10.9)	34.0	2.16, d (10.6) 2.25, td (10.6, 12.5)	33.8
15	3.13, td (12.1, 2.3)	36.4	3.14, td (12.1, 1.7)	36.2	3.13, d (10.6)	34.1
16	-	111.7	-	112.0	-	111.2
17	7.58, s	162.4	7.56, s	162.0	7.53, s	162.2
18	0.75, t (7.5)	10.5	0.76, t (7.5)	9.7	1.10, d (7.0)	16.6
19	1.24, m 1.34, m	24.0	1.14, m 1.34, m	23.7	3.04, m	27.2
20	3.25, t (12.0)	40.9	3.02, m	41.5	2.64, br s	40.6
21	4.55, d (12.0)	67.0	4.39, m	65.8	4.31, br s	89.2
22	3.66, s	51.5	3.75, s	51.4	3.70, s	51.8
23	-	169.1	-	169.3	-	169.9
24	3.98, s	62.3	3.75, s	62.2	3.83, s	62.3
2′	4.91, d (4.5)	81.2	4.84, d (3.4)	79.9	4.90, s	79.7
3′	4.20, t (4.5)	66.6	4.21, t (4.2)	67.3	4.23, ov	67.1
4′a 4′b	2.82, d (16.8) 2.94, dd (16.8, 4.5)	29.3	2.75, d (16.1) 2.90, dd (16.1, 4.2)	29.3	2.73, dd (16.7, 3.5) 2.90, dd (16.7, 4.5)	29.1
5′	-	156.0	-	157.1	-	156.0
6′	6.11, s	96.3	-	101.5	6.03, s	96.1
7′	-	159.1	-	156.6	-	152.4
8′	-	100.6	6.07, s	96.0	-	109.2
9′	-	156.3	-	156.1	-	153.7
10′	-	100.6	-	100.9	-	101.5
1′′	-	131.9	-	132.2	-	132.4
2′′	7.05, ov.	116.0	6.98, ov.	115.4	6.95, d (1.5)	115.4
3′′	-	146.3	-	145.9	-	146.1
4′′	-	146.2	-	146.0	-	145.8
5′′	6.79, d (8.2)	116.0	6.75, d (8.2)	116.0	6.76, d (8.2)	116.0
6′′	6.87, dd (2.4, 8.2)	120.1	6.79, dd (1.9, 8.2)	119.4	6.80, dd (1.5, 8.2)	119.5

*^a^* Recorded at 500/125 MHz, *^b^* Recorded at 400/100 MHz.

## Supplementary Materials

The following are available online. Experimental procedures; 1D and 2D NMR, key ROE correlations, and proposed biosynthesis for **1**–**3** (PDF).
