# Supplementary material for: Corynanthean-Epicatechin Flavoalkaloids from Corynanthe pachyceras
Source: Molecules. 2020 Jun 7;25(11):2654. doi: 10.3390/molecules25112654 (PMC7321195; doi:10.3390/molecules25112654)
Supplement: Supplementary file 1 [file molecules-25-02654-s001.pdf]

Supporting Information Of

Corynanthean-Epicatechin Flavoalkaloids from *Corynanthe pachyceras*

Tapé Kouamé[1,2], Aboua Timothée Okpekon[2], Nicaise F. Bony[3], Amon Diane N'Tamon[1,3], Jean-François Gallard[4], Somia Rharrabti[1], Karine Leblanc[1], Elisabeth Mouray[5], Philippe Grellier[5], Pierre Champy[1], Mehdi A. Beniddir[1,*], and Pierre Le Pogam[1,*]

[1] Université Paris-Saclay, CNRS, BioCIS, 92290, Châtenay-Malabry, France

[2] Laboratoire de Chimie Organique et de Substances Naturelles (LCOSN), UFR Sciences des Structures de la Matière et Technologie, Univ. FHB, 22 BP 582 Abidjan 22, Côte d'Ivoire

[3] Département de Chimie Analytique, Minérale et Générale, Technologie Alimentaire, UFR Sciences Pharmaceutiques et Biologiques, Univ. FHB, 06 B. P. 2256 Abidjan 06, Côte d'Ivoire

[4] Institut de Chimie des Substances Naturelles, CNRS, ICSN UPR 2301, Université Paris-Saclay, 21 Avenue de la Terrasse, 91198 Gif-sur-Yvette, France

[5] Muséum National d'Histoire Naturelle, Unité Molécules de Communication et Adaptation des Micro-organismes, UMR7245, CP54, 57 Rue Cuvier, 75005, Paris, France

N°	Content	Page
S1	Molecular network of the stem bark alkaloid extract of *C. pachyceras*.	S-4
S2	Molecular network of the stem bark ethanolic extract of *C. pachyceras*.	S-5
S3	HRESIMS spectrum of **1**	S-6
S4	^{1}H NMR (CD_3OD, 500 MHz, 283 k) and ^{1}H NMR (CD_3OD, 500 MHz, 300 k) spectrum of **1**	S-7
S5	^{1}H NMR (CD_3OD, 500 MHz, 283 k) spectrum of **1**	S-8
S6	^{13}C NMR (CD_3OD, 125 MHz) spectrum of **1**	S-9
S7	COSY (CD_3OD, 500 MHz) spectrum of **1**	S-10
S8	HSQC (CD_3OD, 500 MHz, 125 MHz) spectrum of **1**	S-11
S9	HMBC (CD_3OD, 500 MHz, 125 MHz) spectrum of **1** (expanded view)	S-12
S10	HMBC (CD_3OD, 500 MHz, 125 MHz) spectrum of **1** (2.6 to 5.3 ppm in ^{1}H dimension)	S-13
S11	ROESY (CD_3OD, 500 MHz) spectrum of **1**	S-14
S12	Key ROE correlations of **1** (A: monoterpene indole alkaloid part; B: epicatechin part)	S-15
S13	HRESIMS spectrum of **2**	S-16
S14	^{1}H NMR (CD_3OD, 400 MHz, 298 K) spectrum of **2**	S-17
S15	^{13}C NMR (CD_3OD, 100 MHz) spectrum of **2**	S-18
S16	COSY (CD_3OD, 400 MHz) spectrum of **2**	S-19
S17	HSQC (CD_3OD, 400 MHz, 100 MHz) spectrum of **2**	S-20
S18	HMBC (CD_3OD, 400 MHz, 100 MHz) spectrum of **2**	S-21
S19	ROESY (CD_3OD, 400 MHz) spectrum of **2**	S-22
S20	^{1}H NMR (DMF-d_7, 600 MHz, 303 K) spectrum of **2**	S-23
S21	^{13}C NMR (DMF-d_7, 150 MHz) spectrum of **2**	S-24
S22	COSY (DMF-d_7, 600 MHz) spectrum of **2**	S-25
S23	HSQC (DMF-d_7, 600 MHz, 150 MHz) spectrum of **2** dimension	S-26
S24	HMBC (DMF-d_7, 600 MHz, 150 MHz) spectrum of **2**	S-27
S25	ROESY (DMF-d_7, 600 MHz) spectrum of **2**	S-28
S26	Key ROE correlations of **2** (A: monoterpene indole alkaloid part; B: epicatechin part)	S-29
S27	HRESIMS spectrum of **3**	S-30
S28	^{1}H NMR (CD_3OD, 500 MHz, 318 K) spectrum of **3**	S-31
S29	^{13}C NMR (CD_3OD, 125 MHz) spectrum of **3**	S-32
S30	COSY (CD_3OD, 500 MHz) spectrum of **3**	S-33
S31	COSY (CD_3OD, 500 MHz) spectrum of **3** (1.6 to 3.9 ppm in ^{1}H dimension)	S-34
S32	HSQC (CD_3OD, 500 MHz, 125 MHz) spectrum of **3** dimension	S-35
S33	HMBC (CD_3OD, 500 MHz, 125 MHz) spectrum of **3**	S-36
S34	HMBC (CD_3OD, 500 MHz, 125 MHz) spectrum of **3** (2.6 to 6.2 ppm in ^{1}H dimension)	S-37
S35	ROESY (CD_3OD, 500 MHz) spectrum of **3**	S-38
S36	ROESY (CD_3OD, 500 MHz) spectrum of **3** (expanded view)	S-39

S37	Key ROE correlations of **3** (A: monoterpene indole alkaloid part; B: epicatechin part)	S-40
S38	Possible planar structures of epicatechocorynantheidine instigating two distinct linkages between the indolomonoterpenic alkaloid component and epicatechin	S-41
S39	Proposed biosynthetic scenario to **1**, **2** and **3**	S-42
S40	HMBC correlation data of compounds **1**-**3**	S-43
S41	GNPS Library Spectrum Accession Codes of the Isolated Compounds	S-44

Figure S1. Molecular network of the stem bark alkaloid extract of *C. pachyceras* highlighting dereplicated compounds against GNPS libraries and targeted nodes for isolation and structure elucidation. This network is accessible at the following address: https://gnps.ucsd.edu/ProteoSAFe/status.jsp?task=0181cbf29f19403e82b44de7ff4654f4.

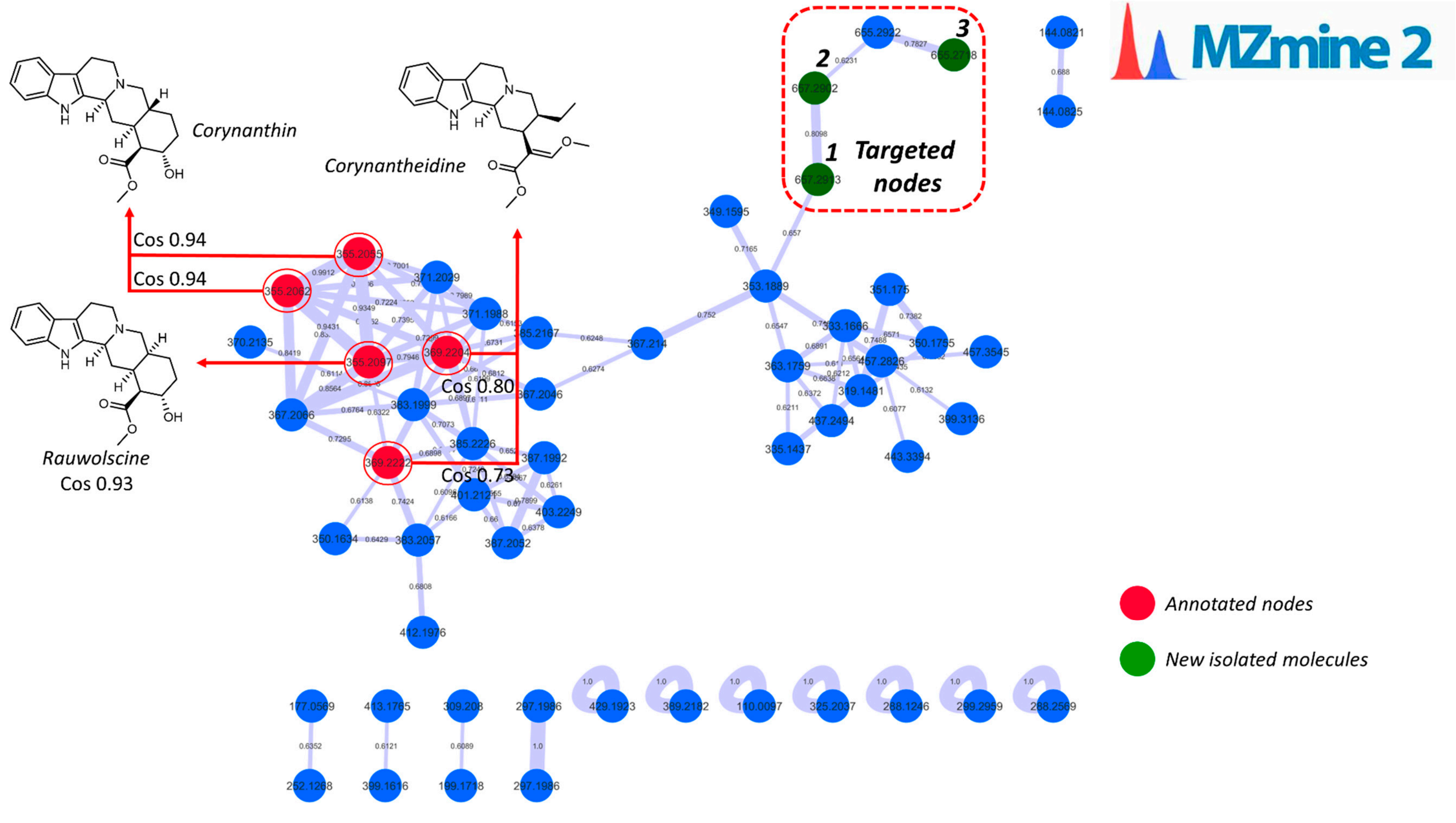

Figure S2. Molecular network of the stem bark ethanolic extract of *C. pachyceras* highlighting dereplicated compounds against GNPS libraries and targeted nodes for isolation and structure elucidation. This network is accessible at the following address: https://gnps.ucsd.edu/ProteoSAFe/status.jsp?task=c7529c8be955485dbbf23ba895a7fa87.

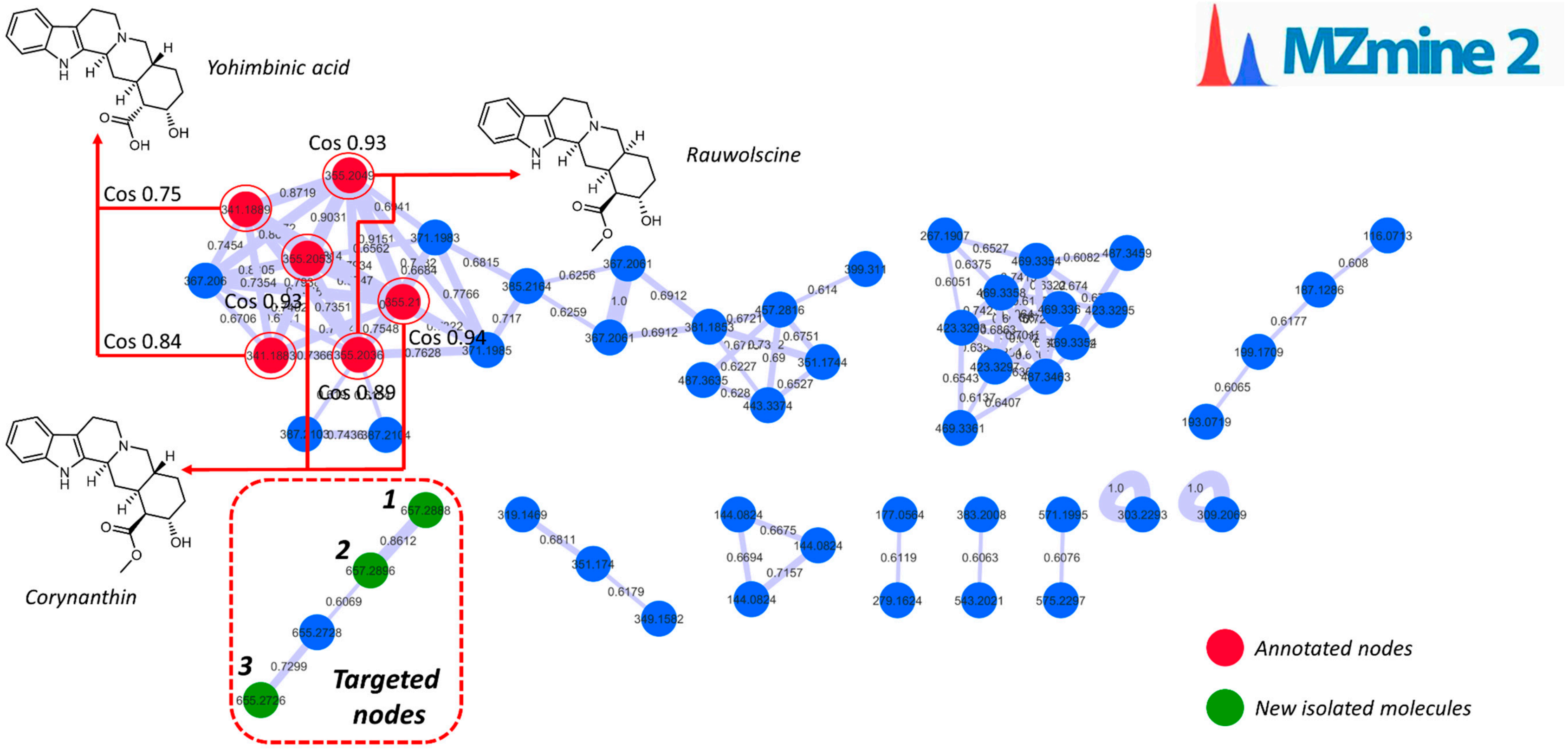

S3. HRESIMS spectrum of **1**

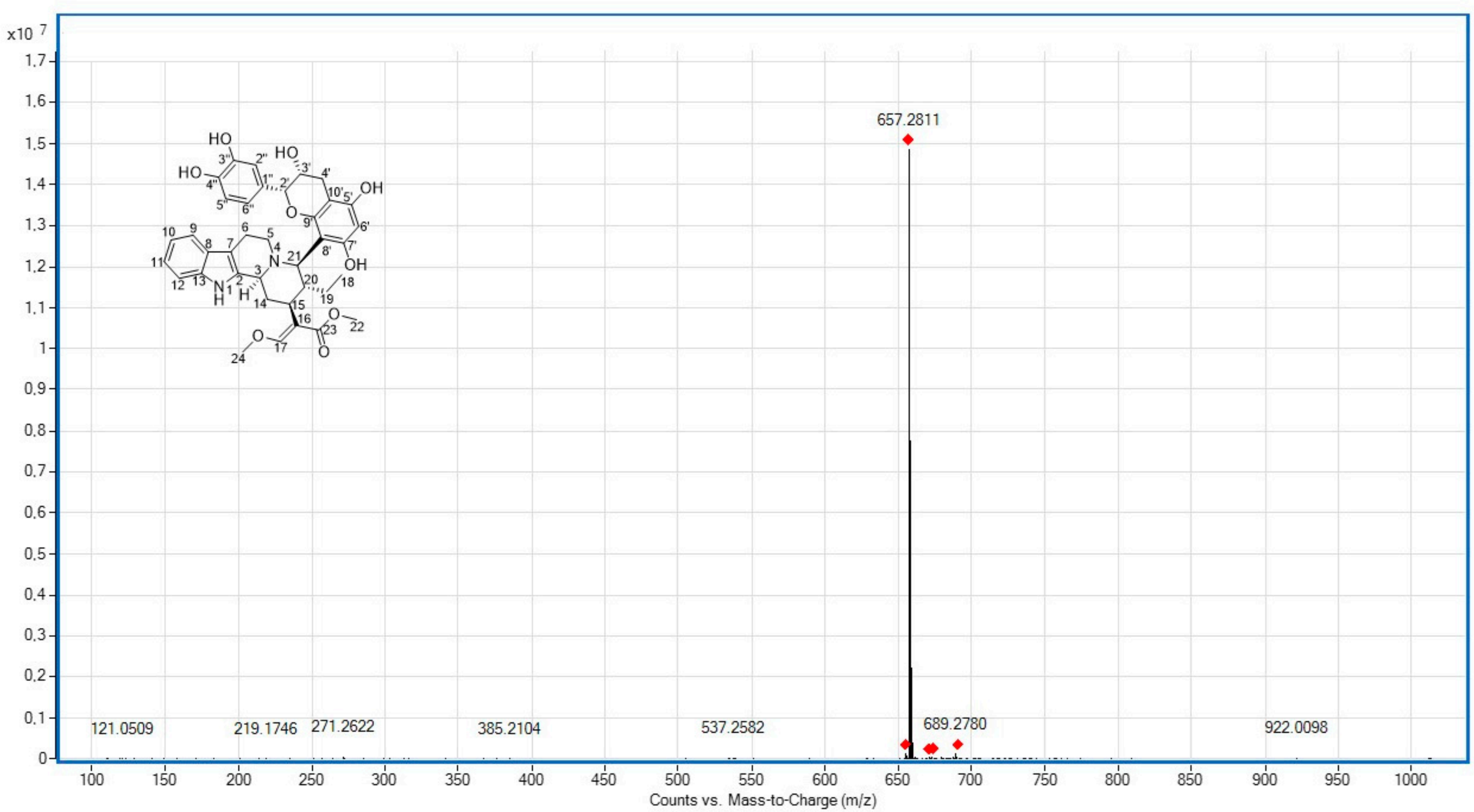

S4. ^{1}H NMR (CD_3OD, 500 MHz, 283 K) and ^{1}H NMR (CD_3OD, 500 MHz, 300 K) spectrum of **1**

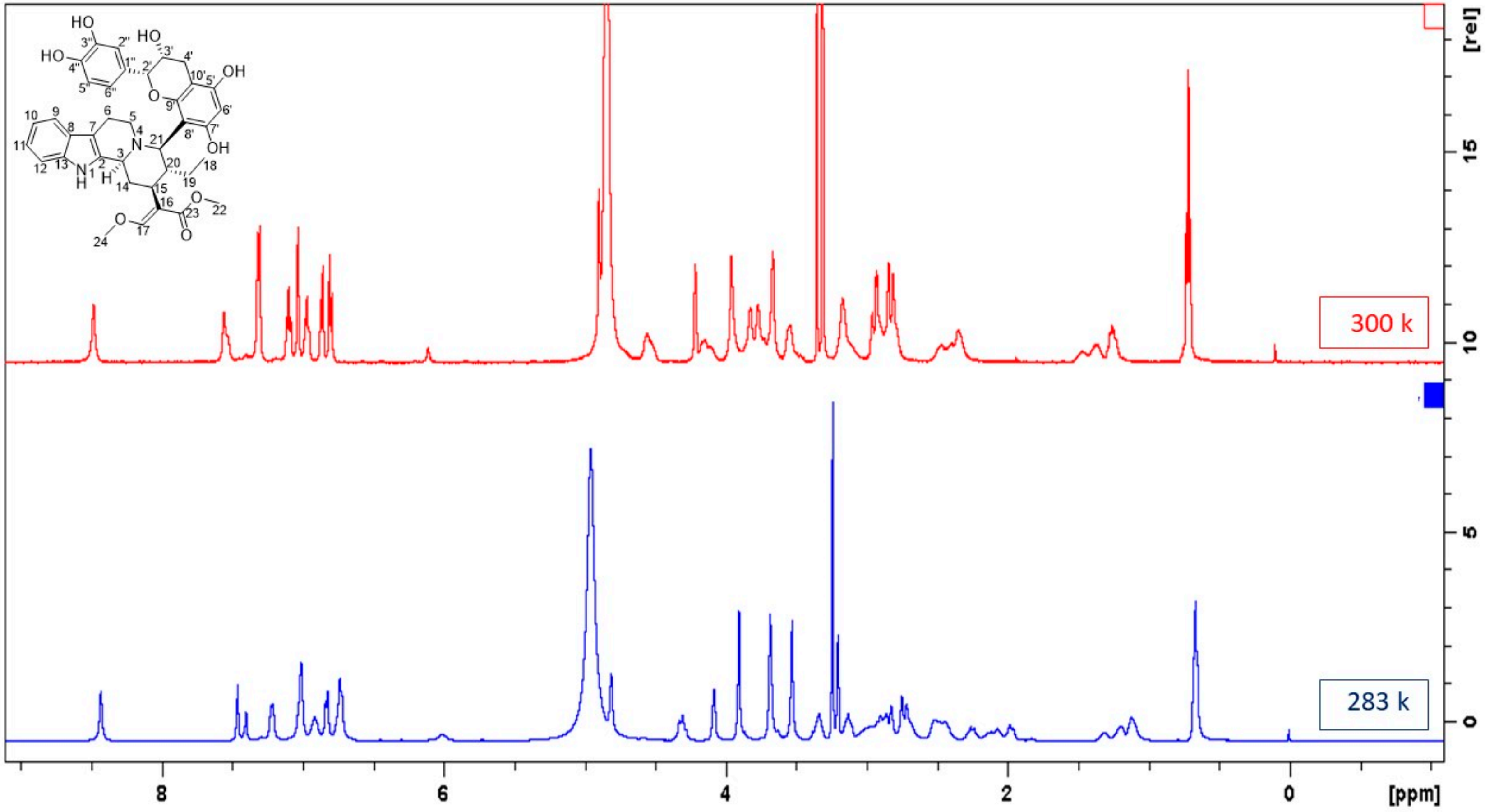

S5. ^{1}H NMR (CD_3OD, 500 MHz, 283 K) spectrum of **1**

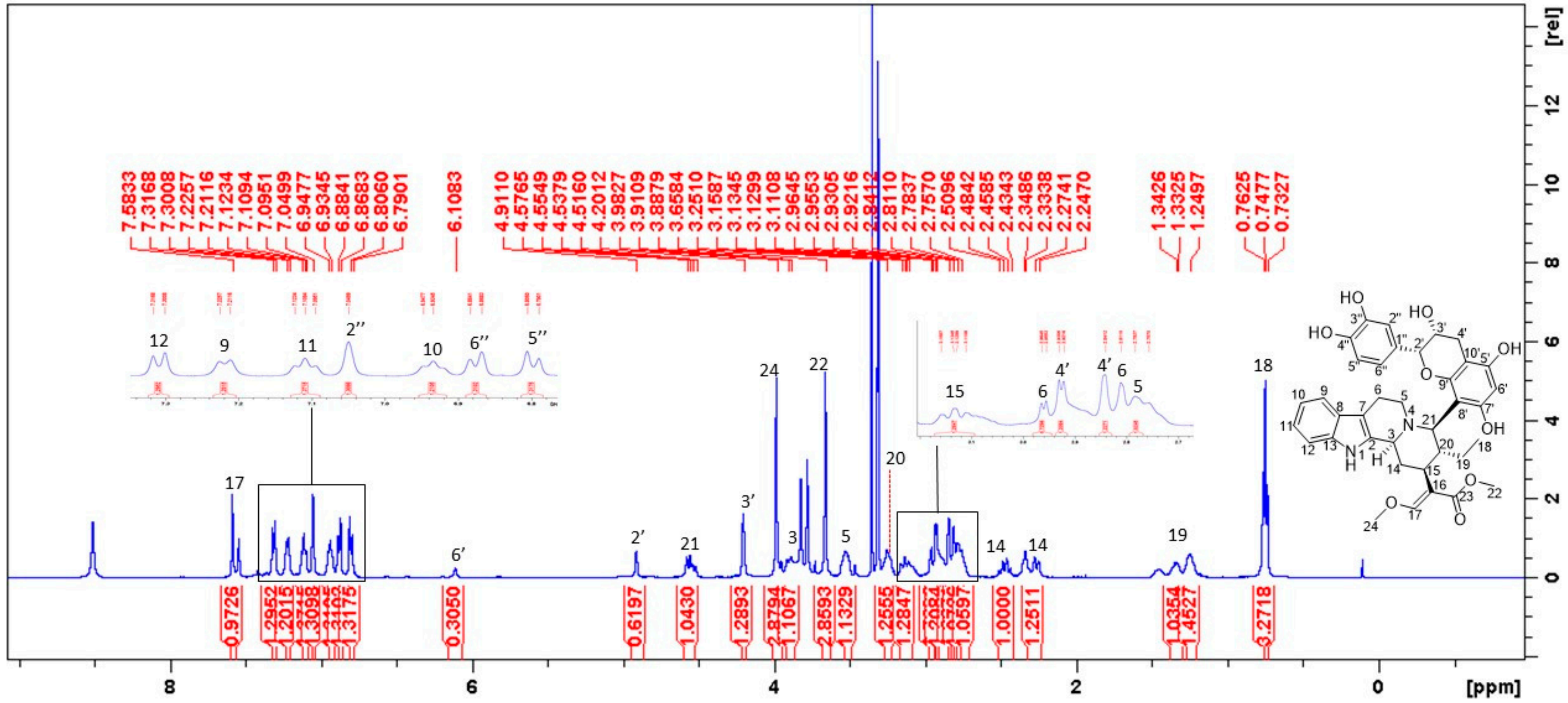

S6. ^{13}C NMR (CD_3OD, 125 MHz) spectrum of **1**

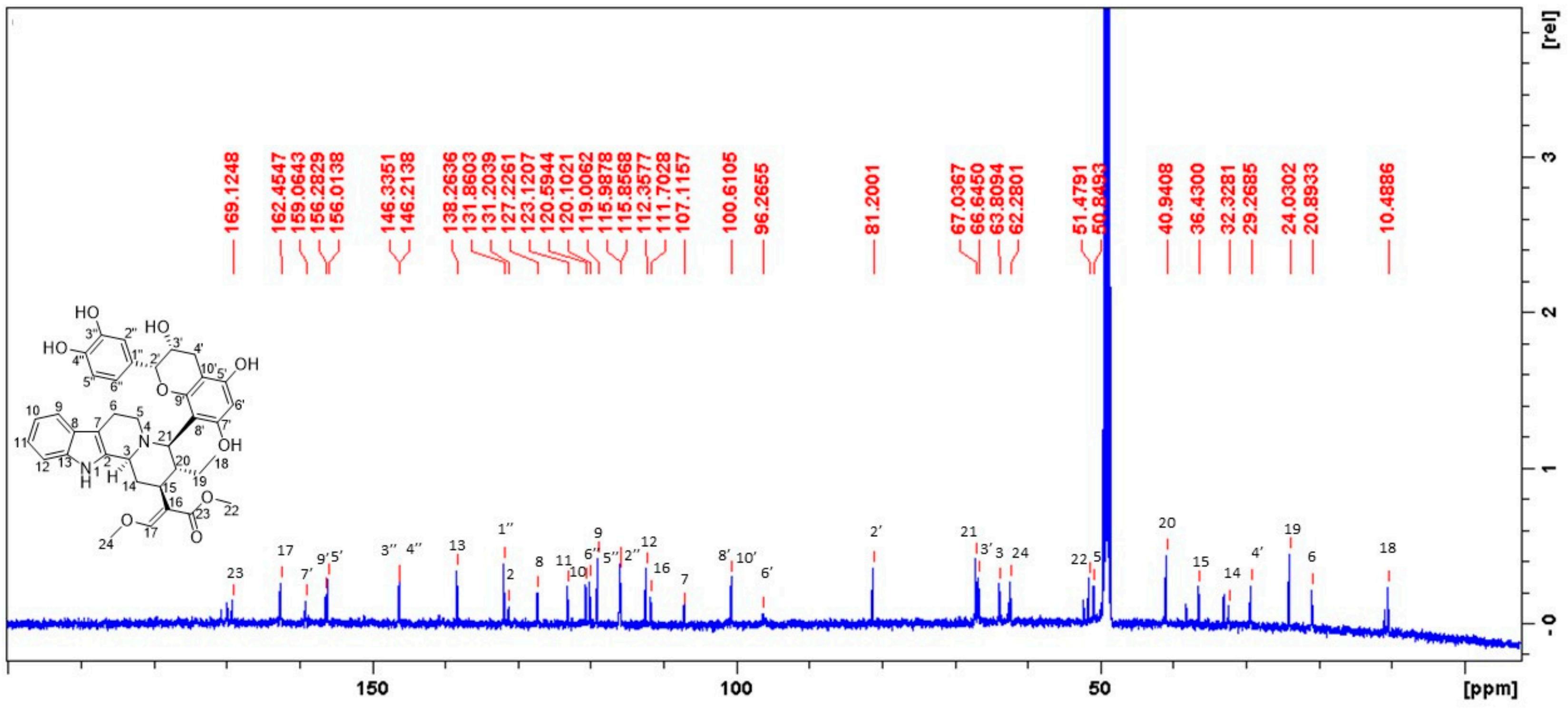

S7. COSY (CD_3OD, 500 MHz) spectrum of **1**

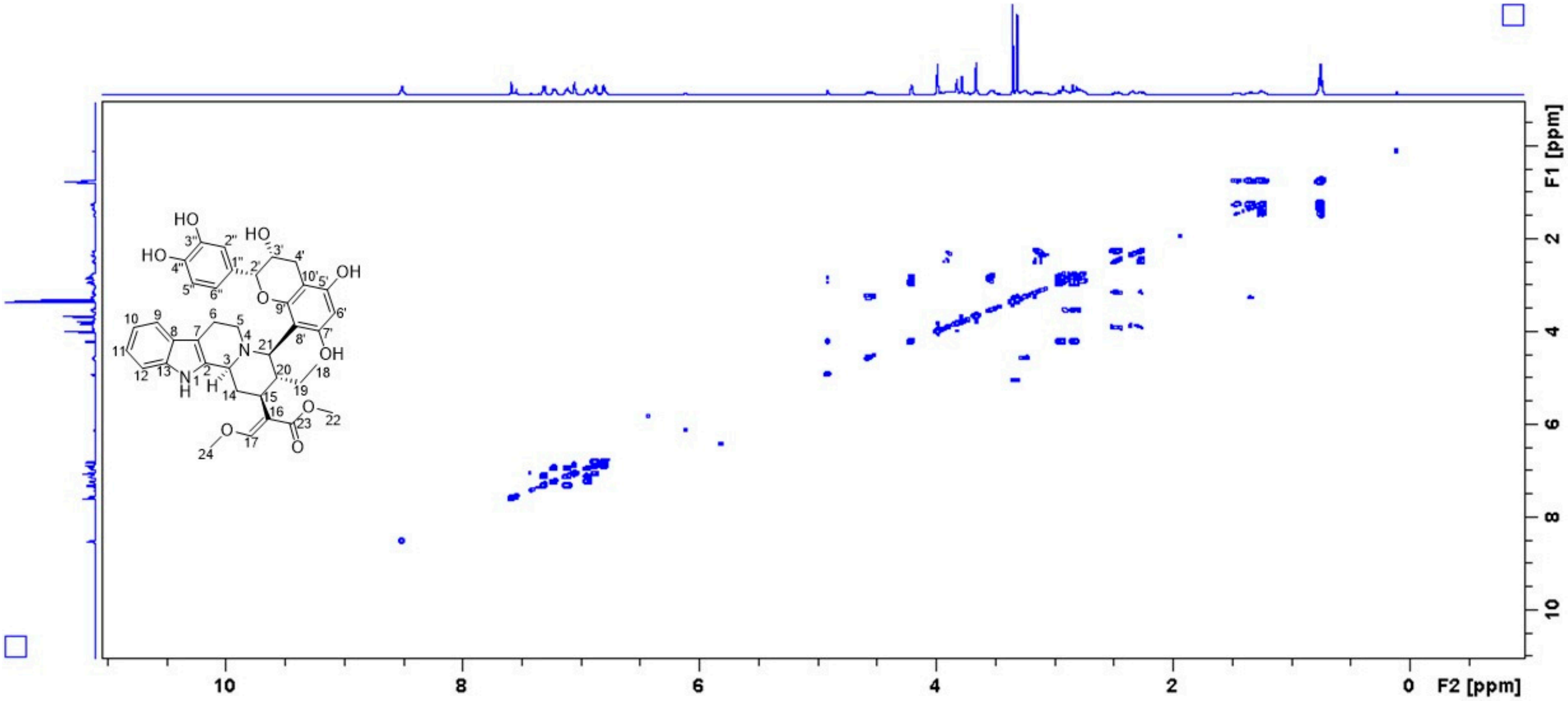

S8. HSQC (CD_3OD, 500 MHz, 125 MHz) spectrum of **1**

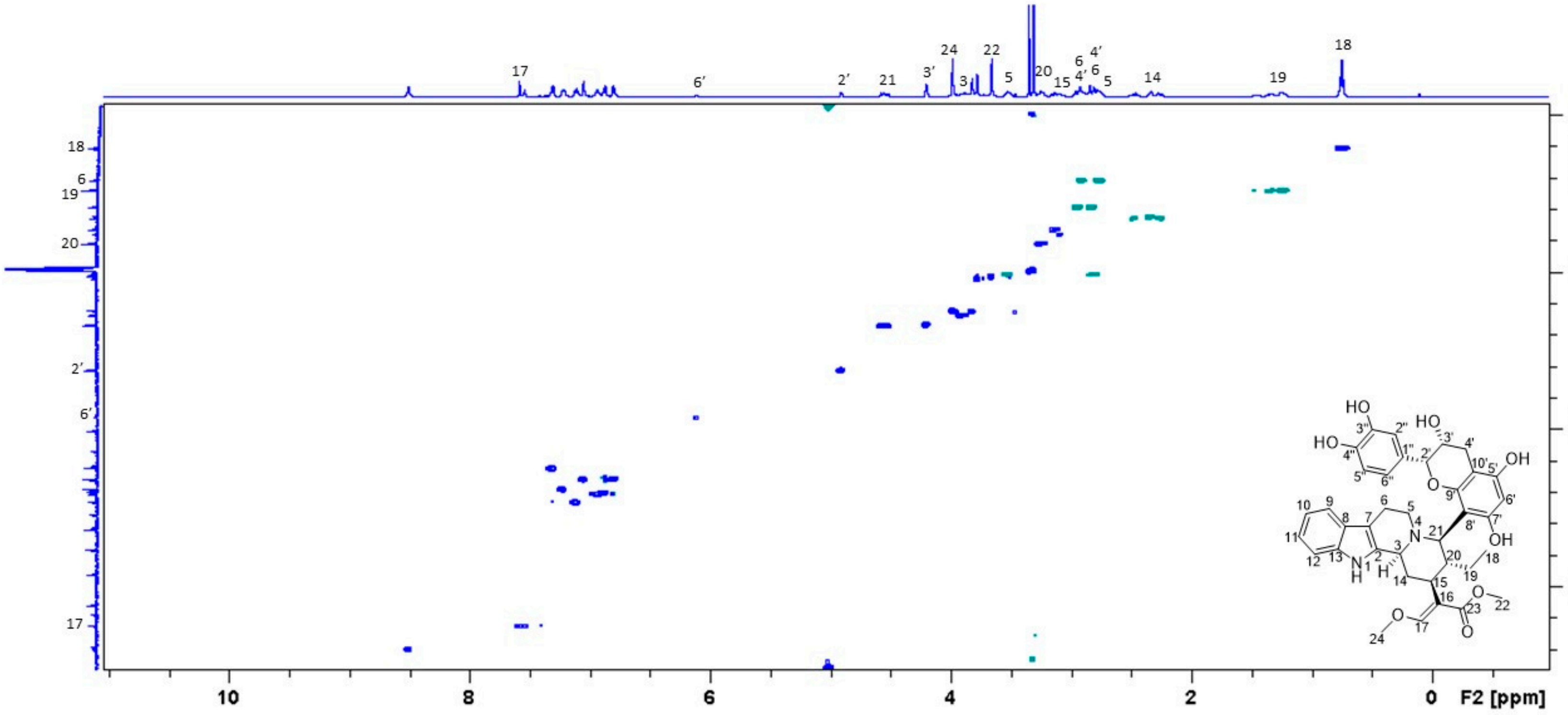

S9. HMBC (CD_3OD, 500 MHz, 125 MHz) spectrum of **1** (expanded view)

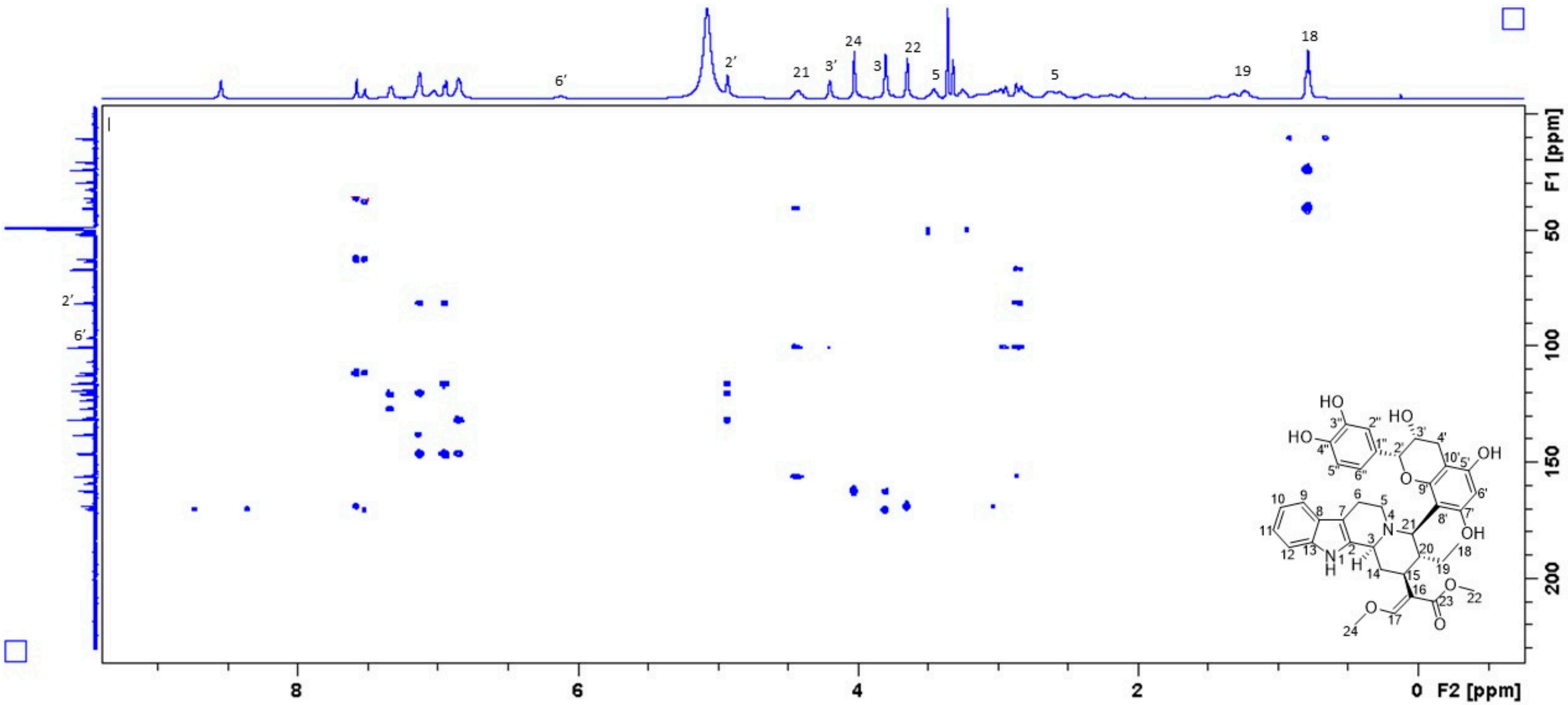

S10. HMBC (CD_3OD, 500 MHz, 125 MHz) spectrum of **1** (2.6 to 5.3 ppm in ^1H dimension)

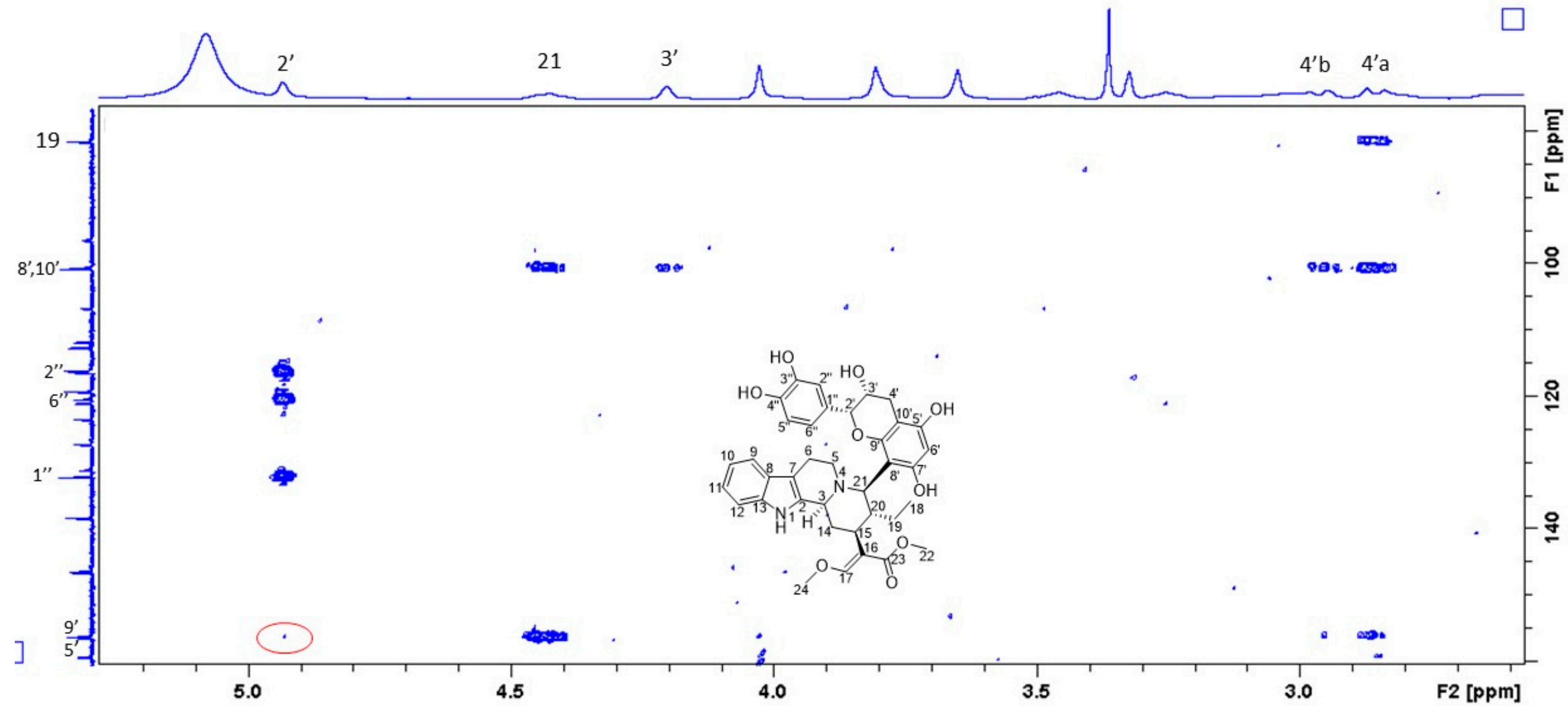

S11. ROESY (CD_3OD, 500 MHz) spectrum of **1**

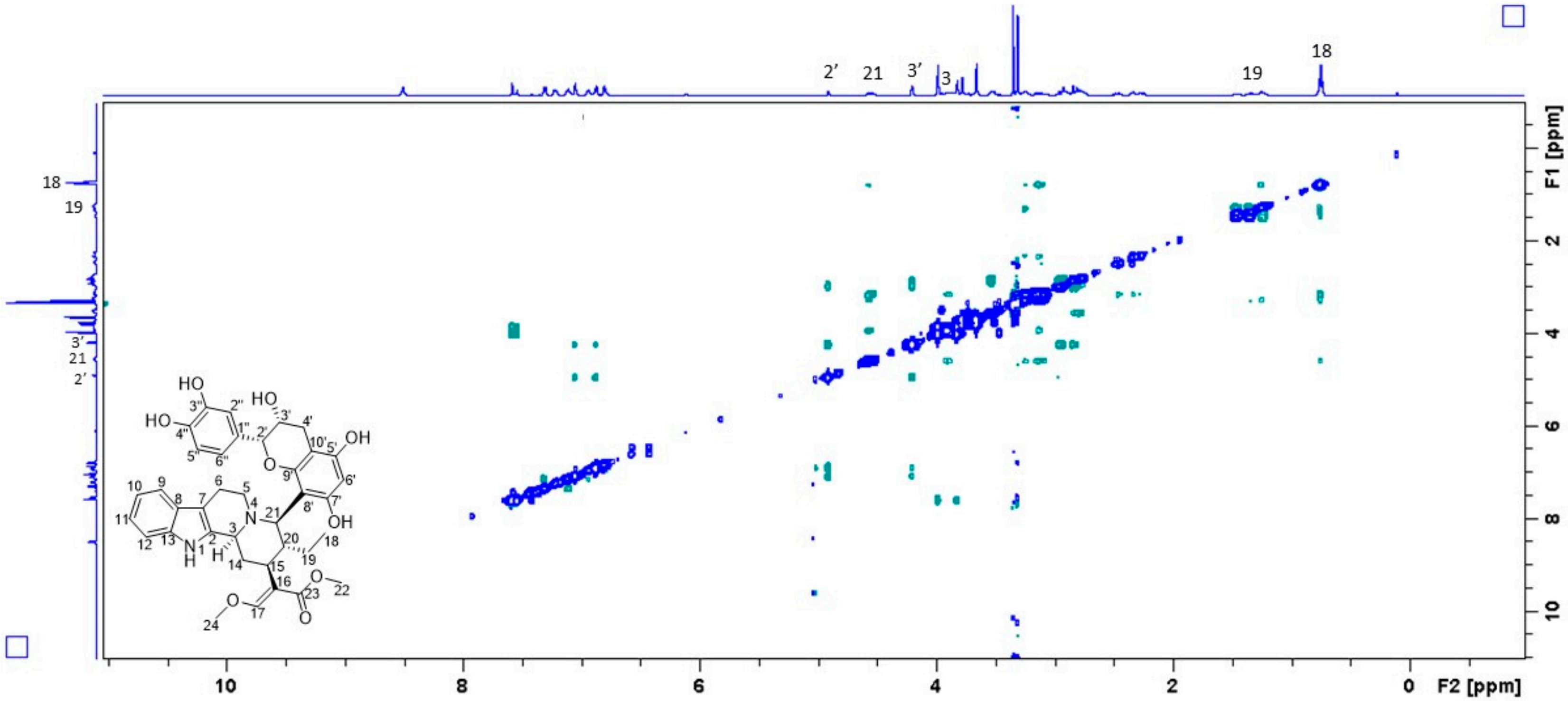

S12. Key ROE correlations of **1** (A: monoterpene indole alkaloid part; B: epicatechin part).

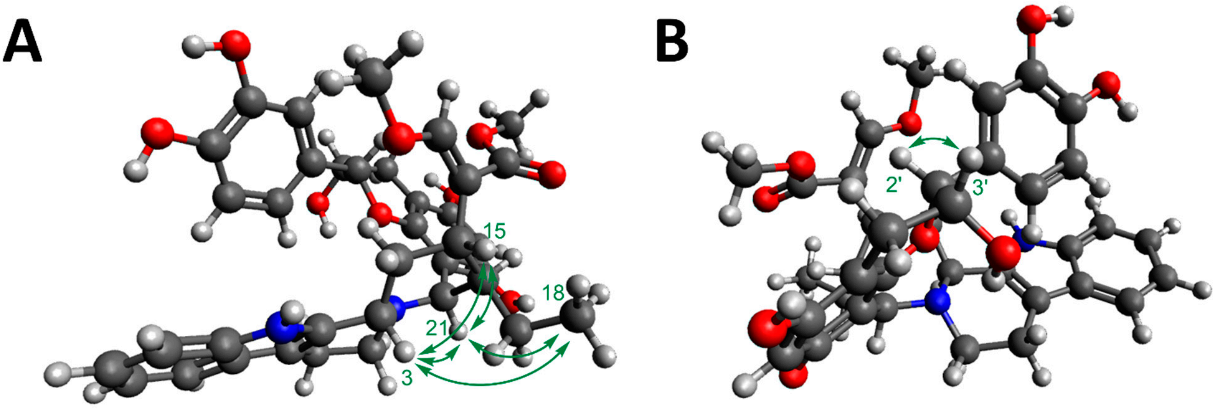

S13. HRESIMS spectrum of **2**

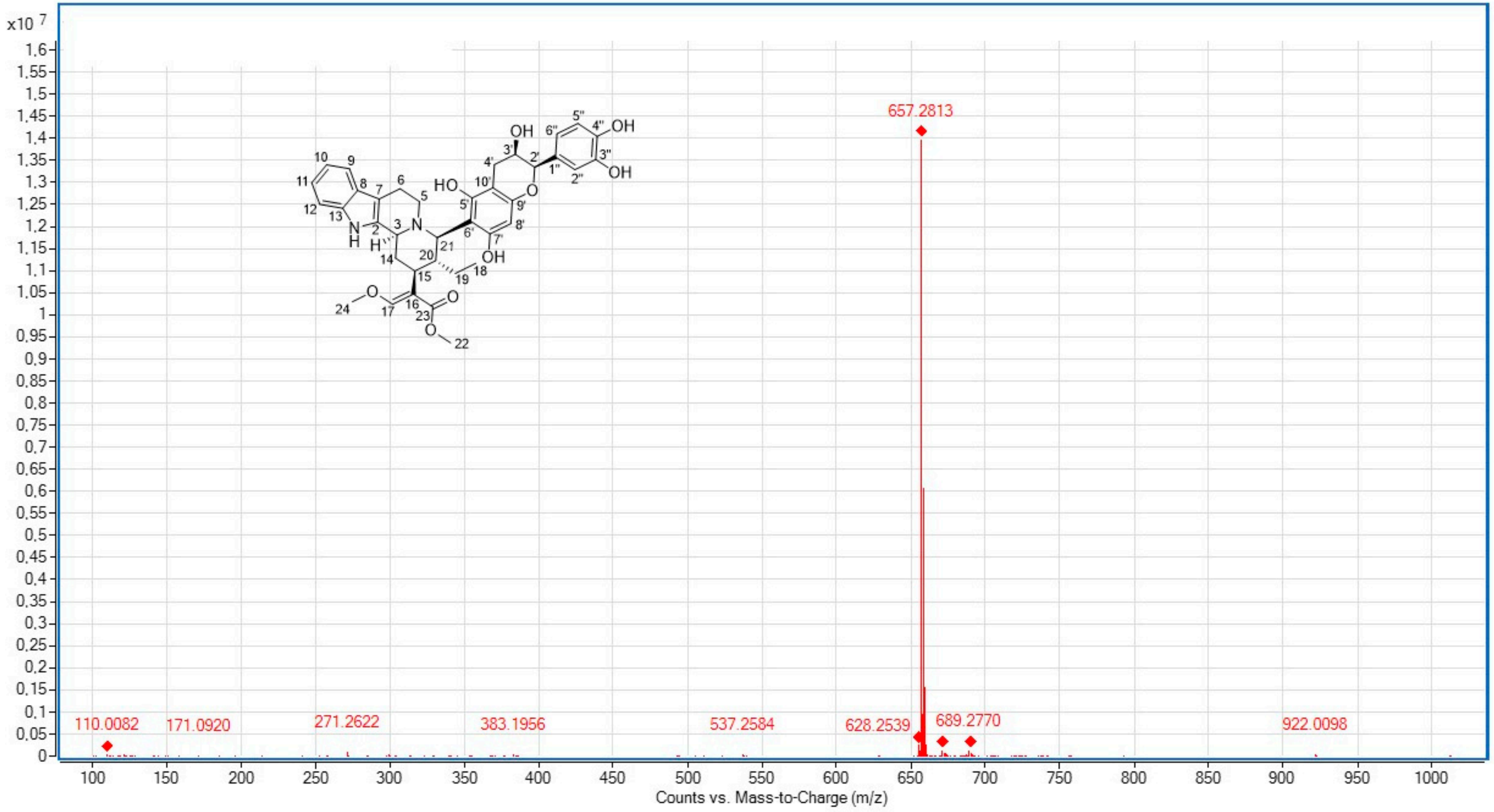

S14. ^{1}H NMR (CD_3OD, 400 MHz, 298 K) spectrum of **2**

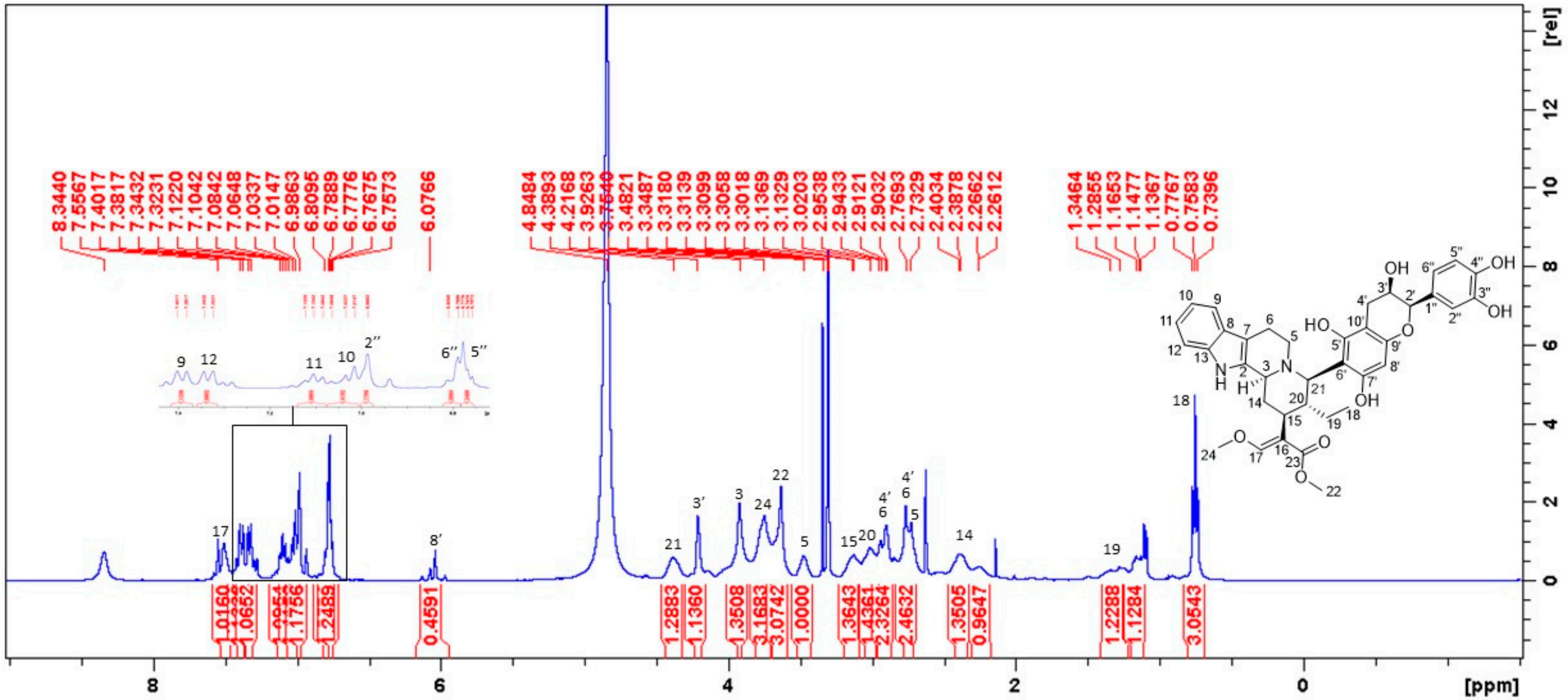

S15. ^{13}C NMR (CD_3OD, 100 MHz) spectrum of **2**

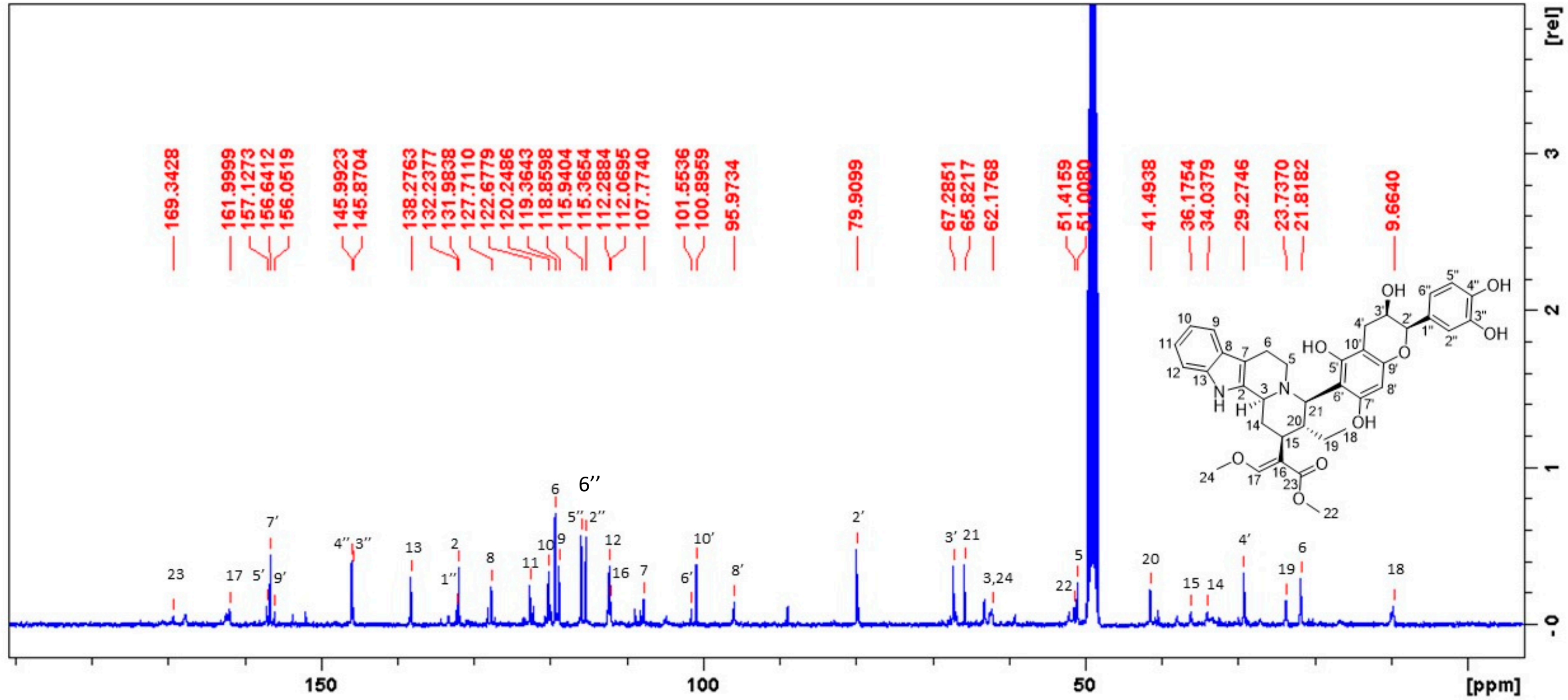

S16. COSY (CD_3OD, 400 MHz) spectrum of **2**

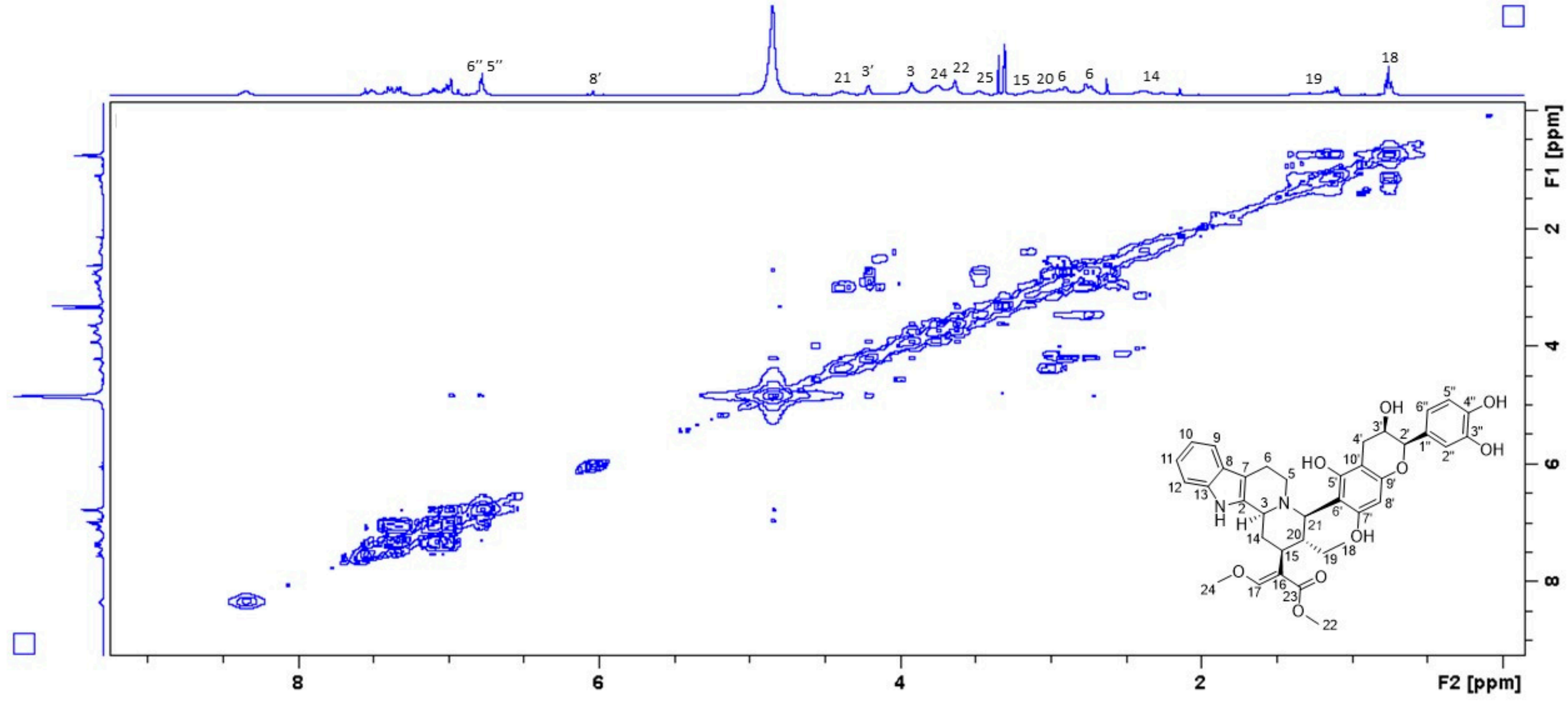

S17. HSQC (CD_3OD, 400 MHz, 100 MHz) spectrum of **2**

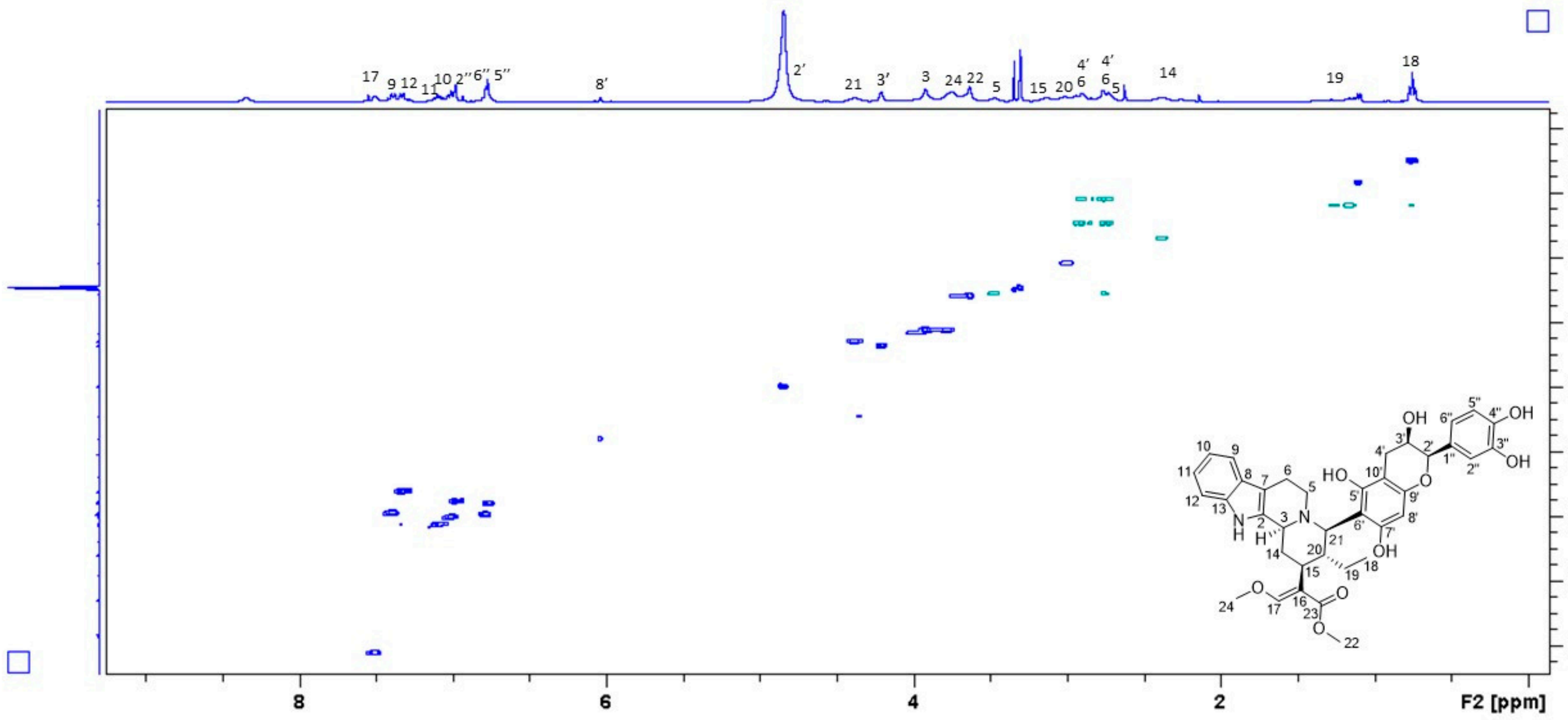

S18. HMBC (CD_3OD, 400 MHz, 100 MHz) spectrum of **2** (expanded view)

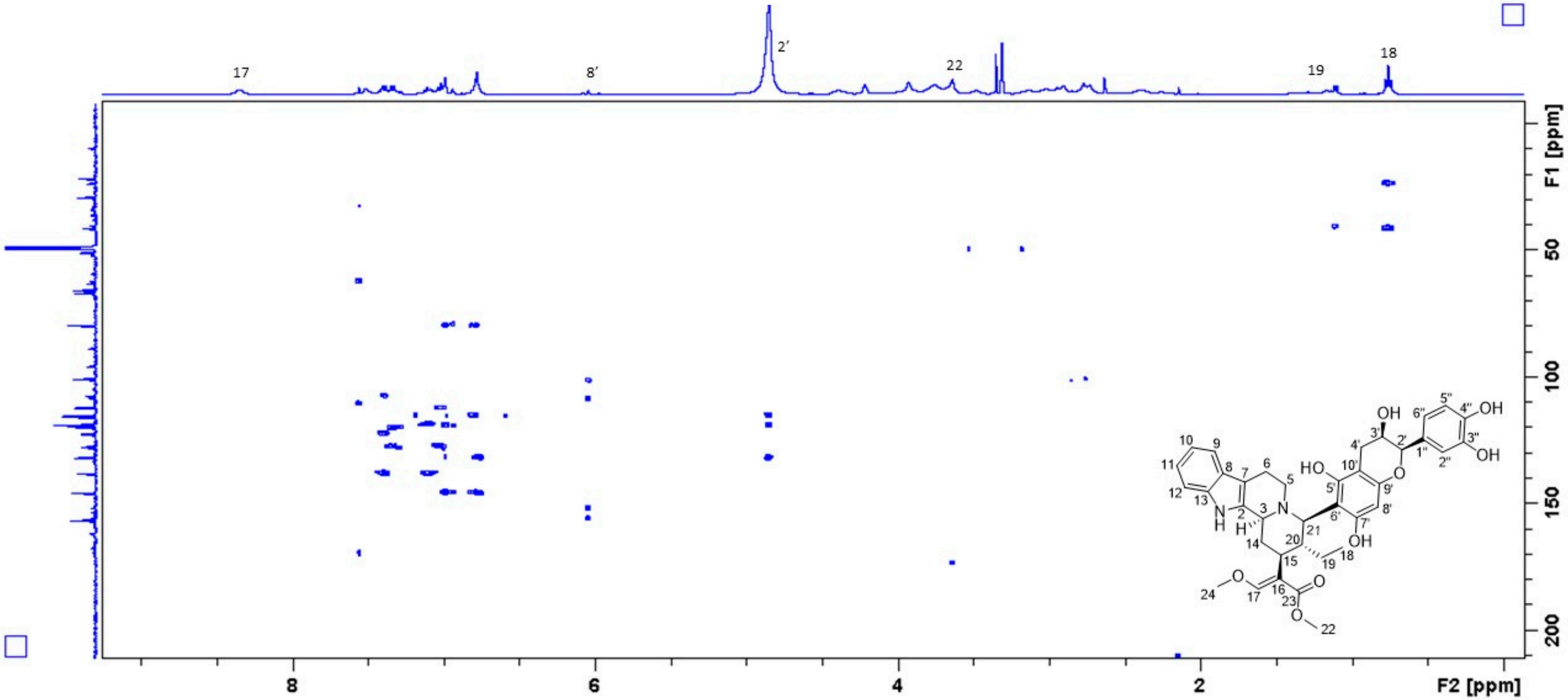

S19. NOESY (CD_3OD, 400 MHz) spectrum of **2**

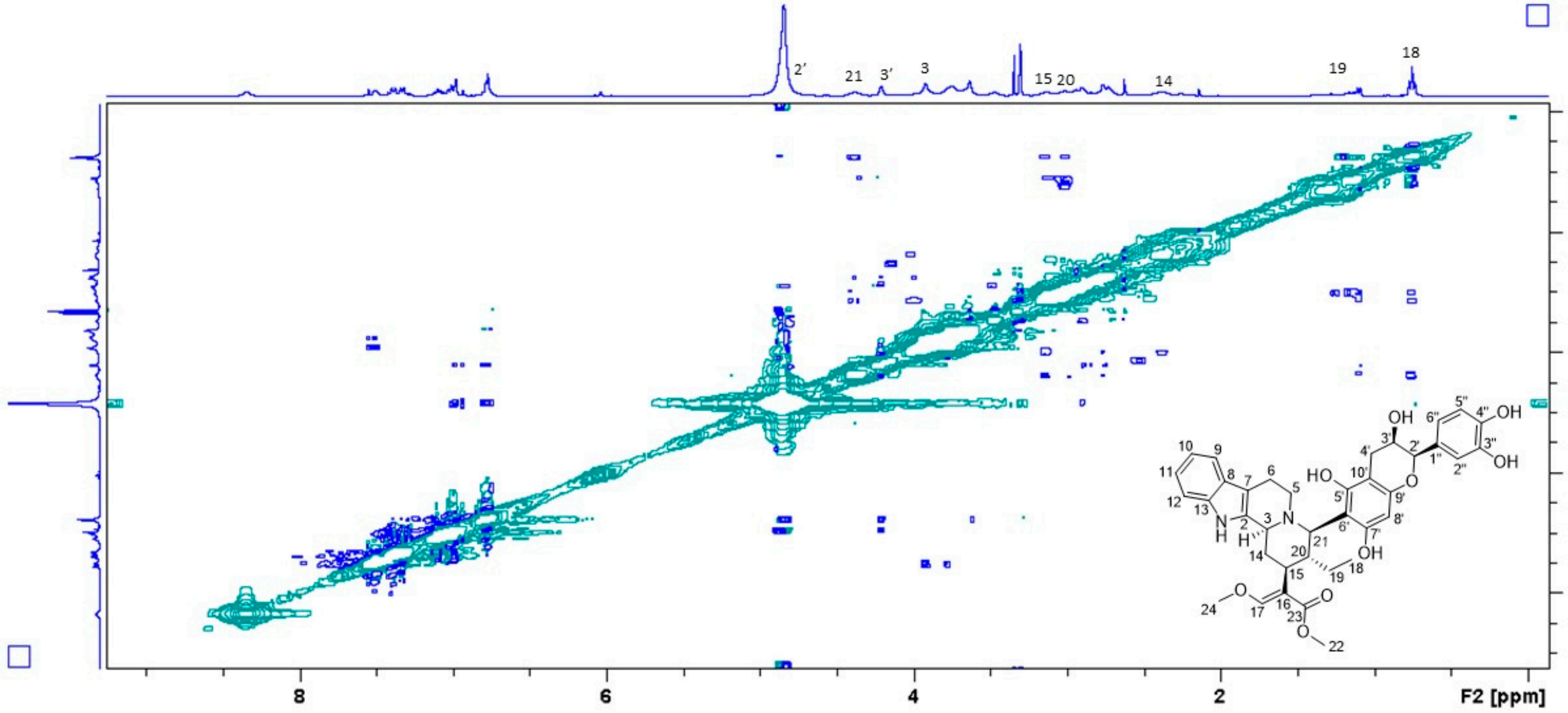

S20. ^{1}H NMR (DMF-d_7, 600 MHz, 303 K) spectrum of **2**

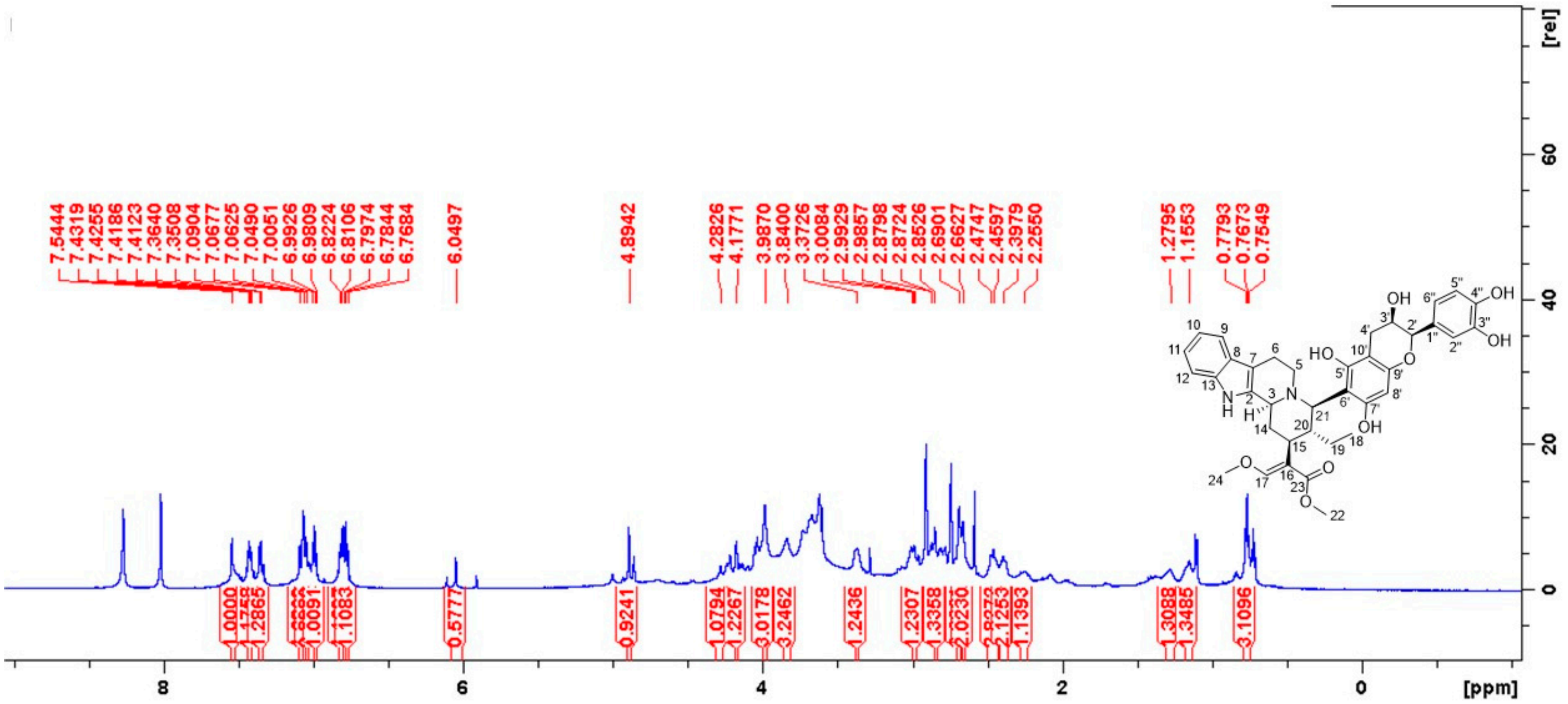

S21. ^{13}C NMR (DMF-d_7, 150 MHz) spectrum of **2**

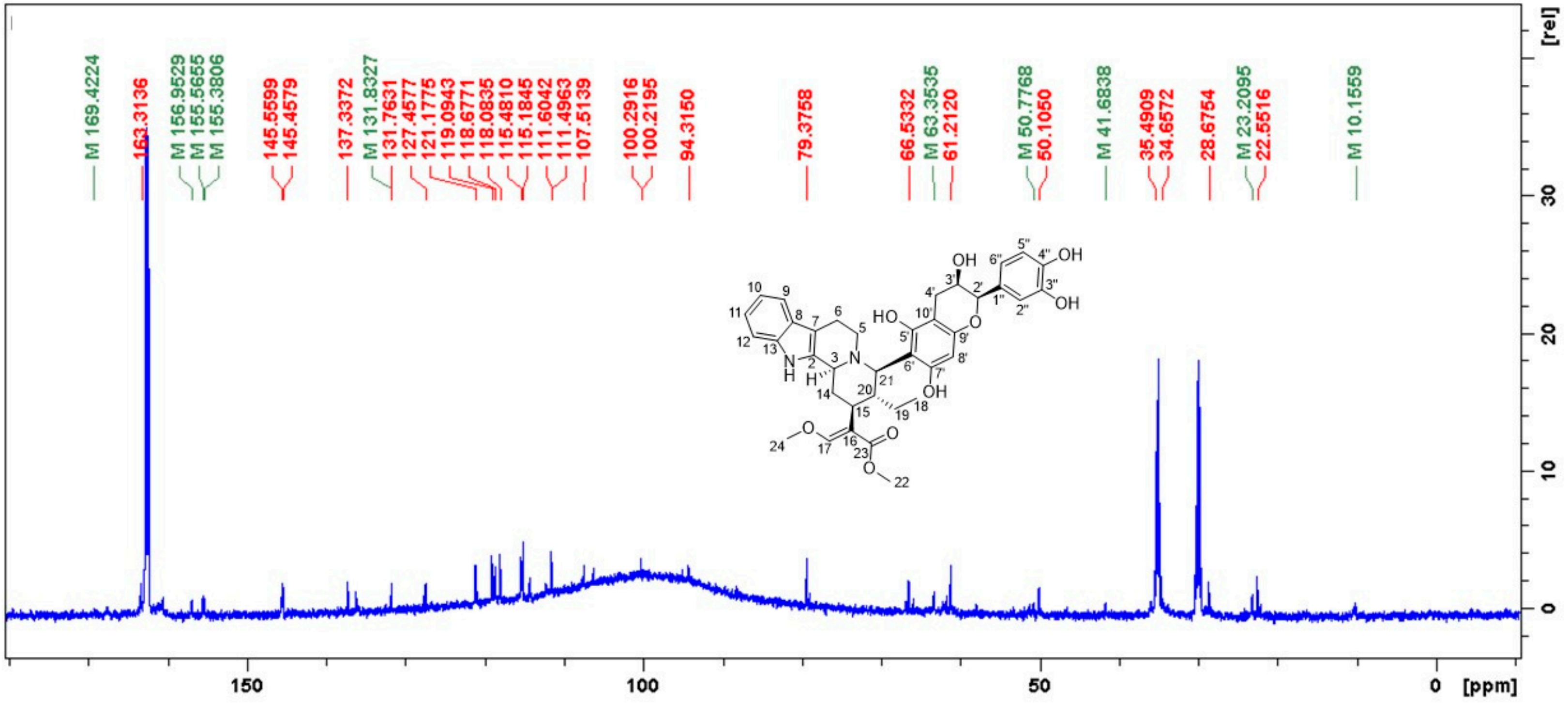

S22. COSY (DMF-d_7, 600 MHz) spectrum of **2**

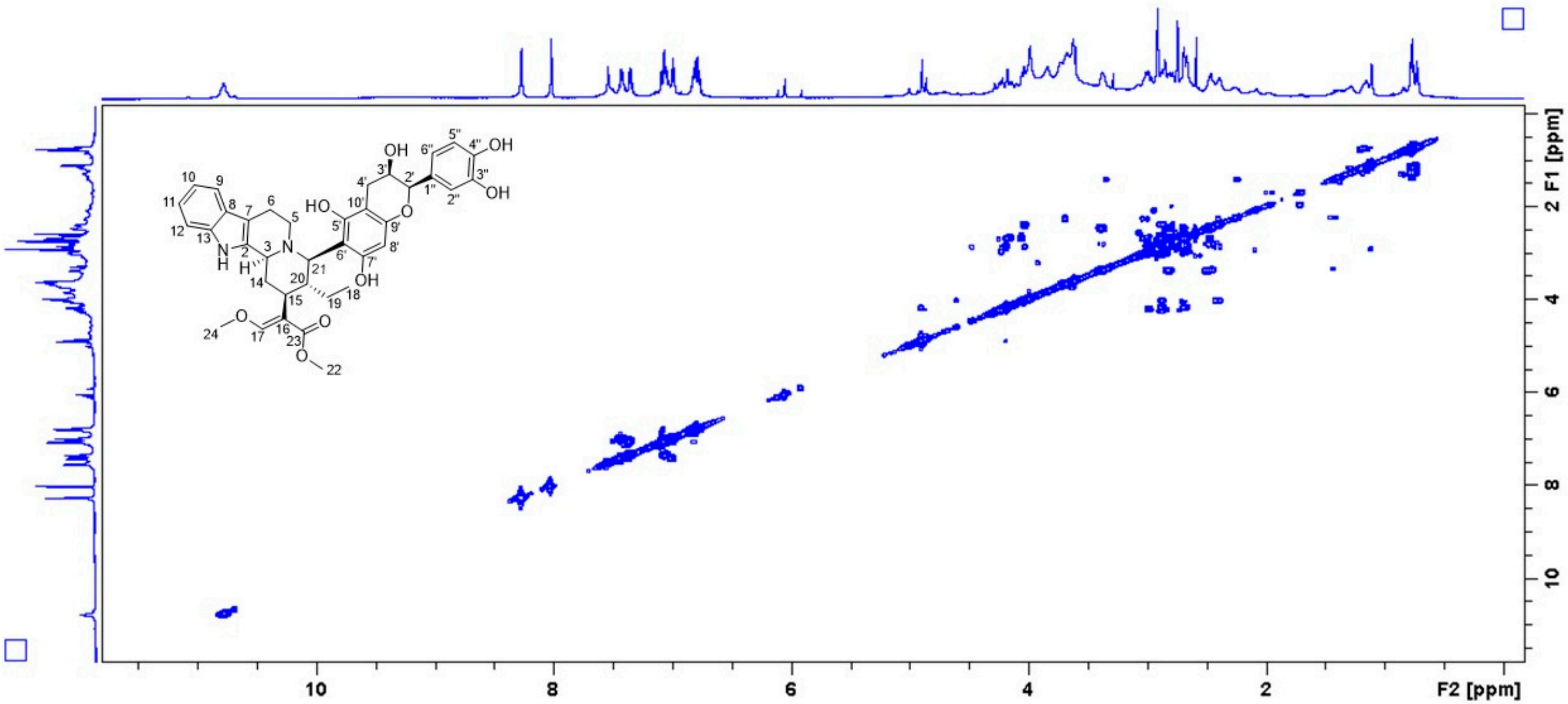

S23. HSQC (DMF-d_7, 600 MHz, 150 MHz) spectrum of **2**

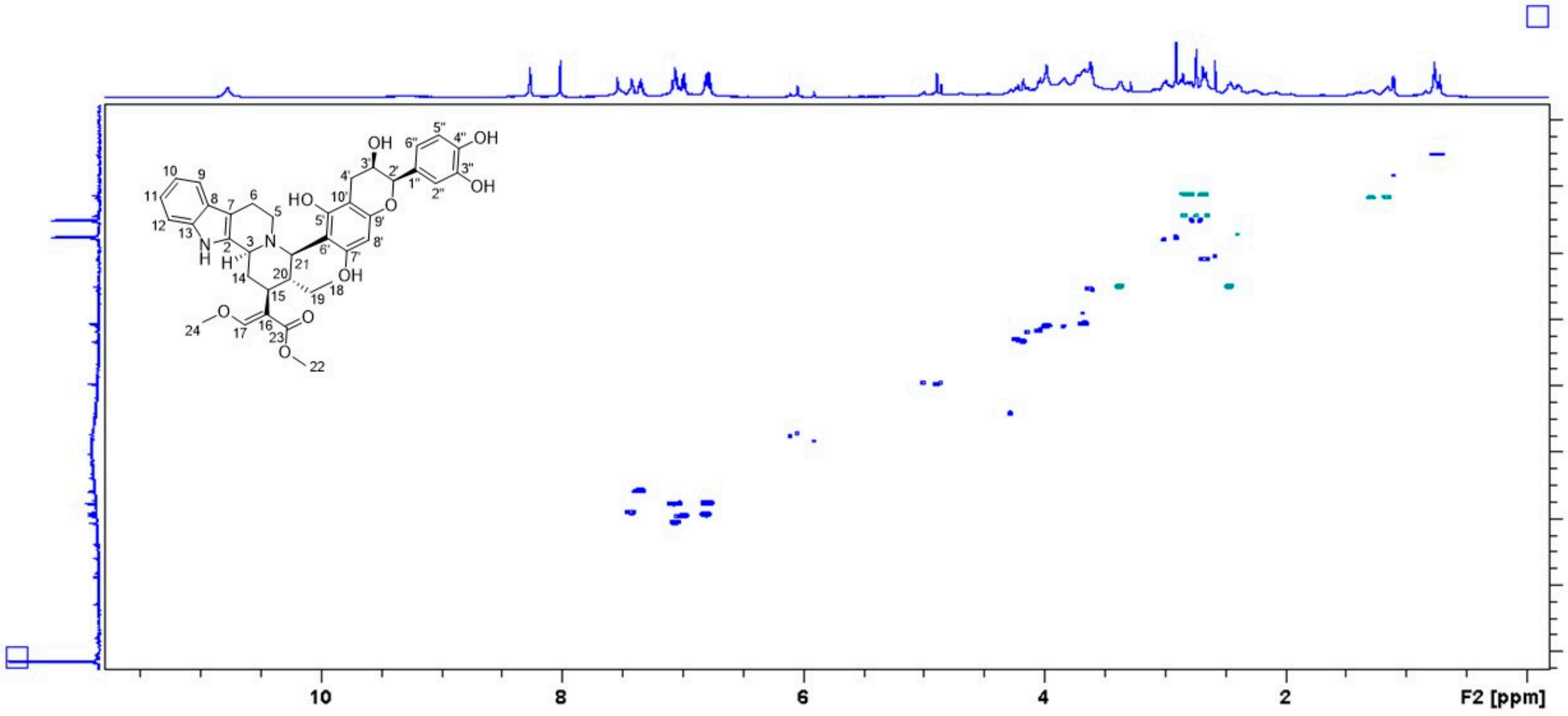

S24. HMBC (DMF-d_7, 600 MHz, 150 MHz) spectrum of **2**

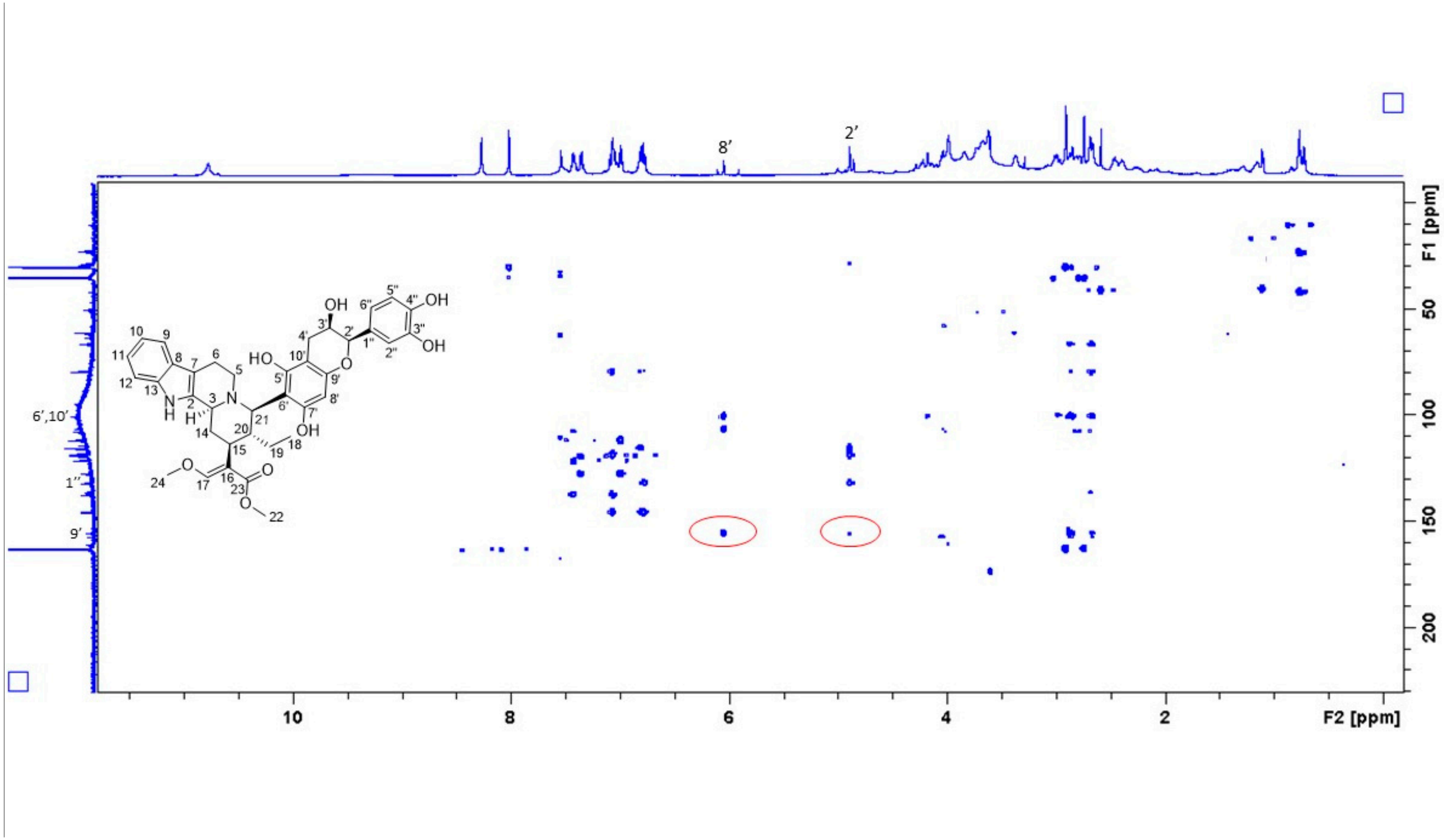

S25. ROESY (DMF-d_7, 600 MHz) spectrum of **2**

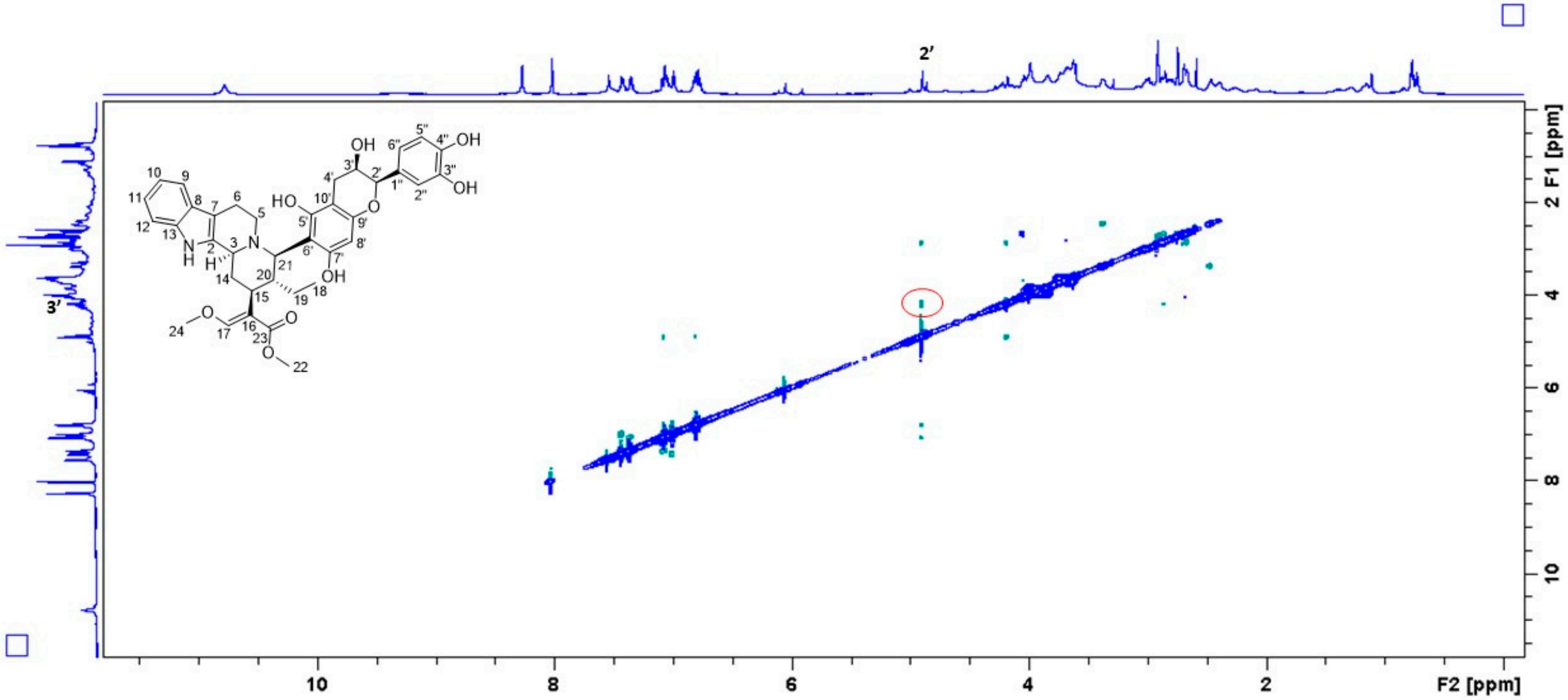

S26. Key ROE correlations of **2** (A: monoterpene indole alkaloid part; B: epicatechin part).

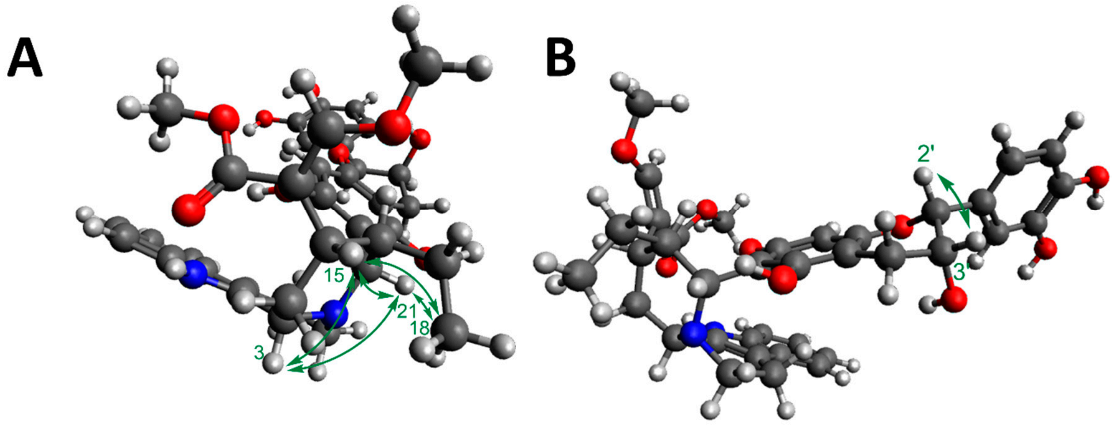

S27. HRESIMS spectrum of **3**

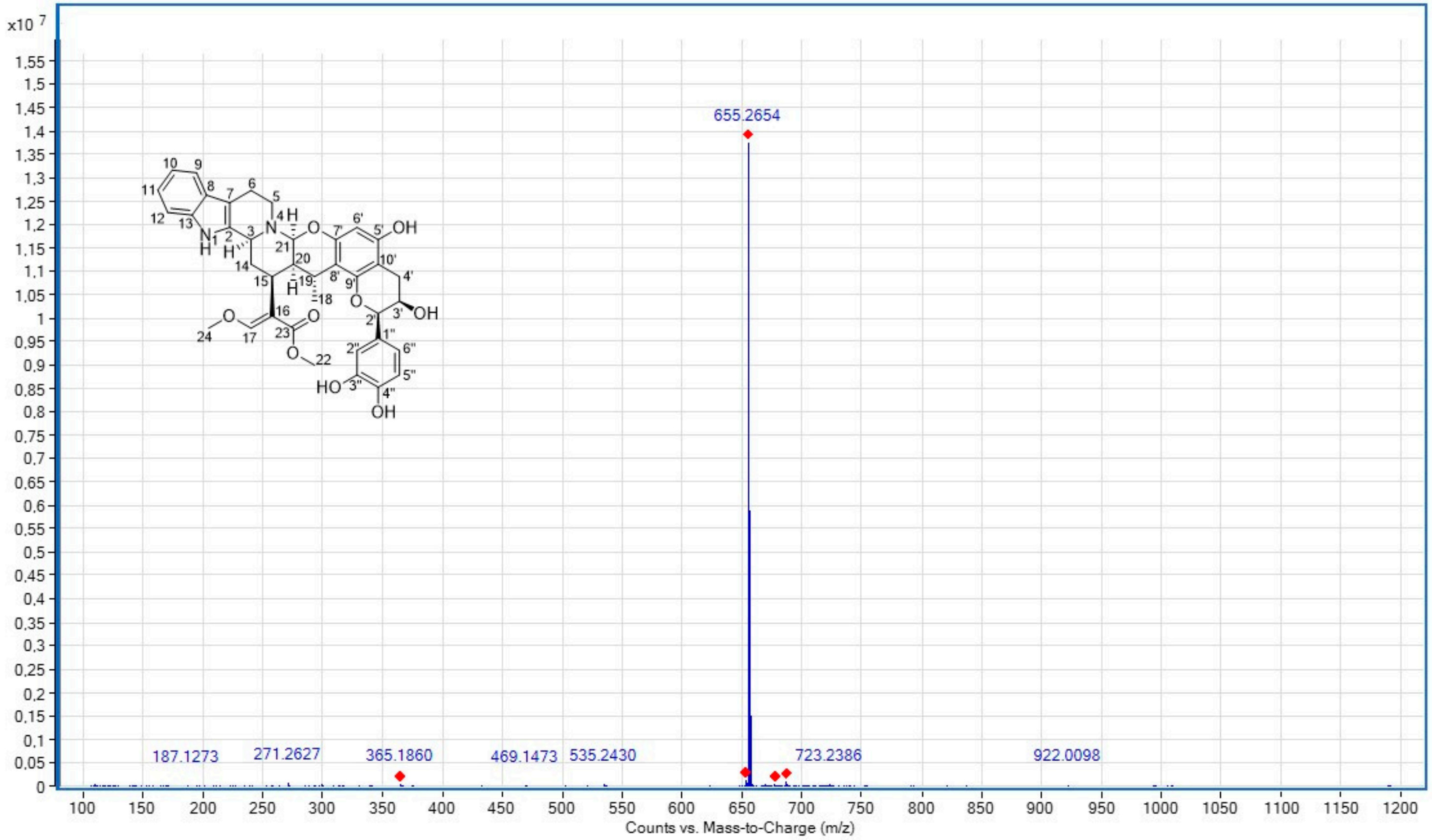

S28. ^{1}H NMR (CD_3OD, 500 MHz, 318 K) spectrum of **3**

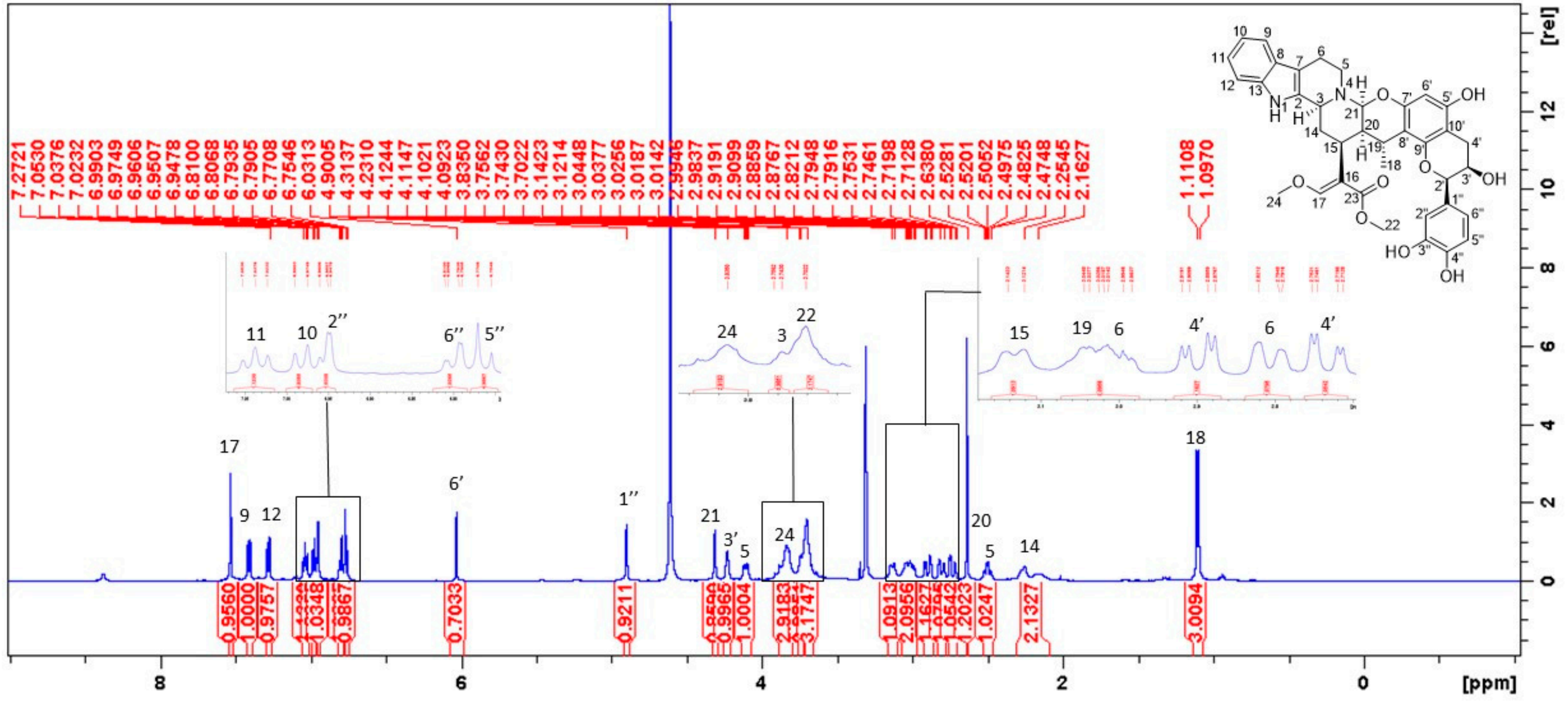

S29. ^{13}C NMR (CD_3OD, 125 MHz) spectrum of **3**

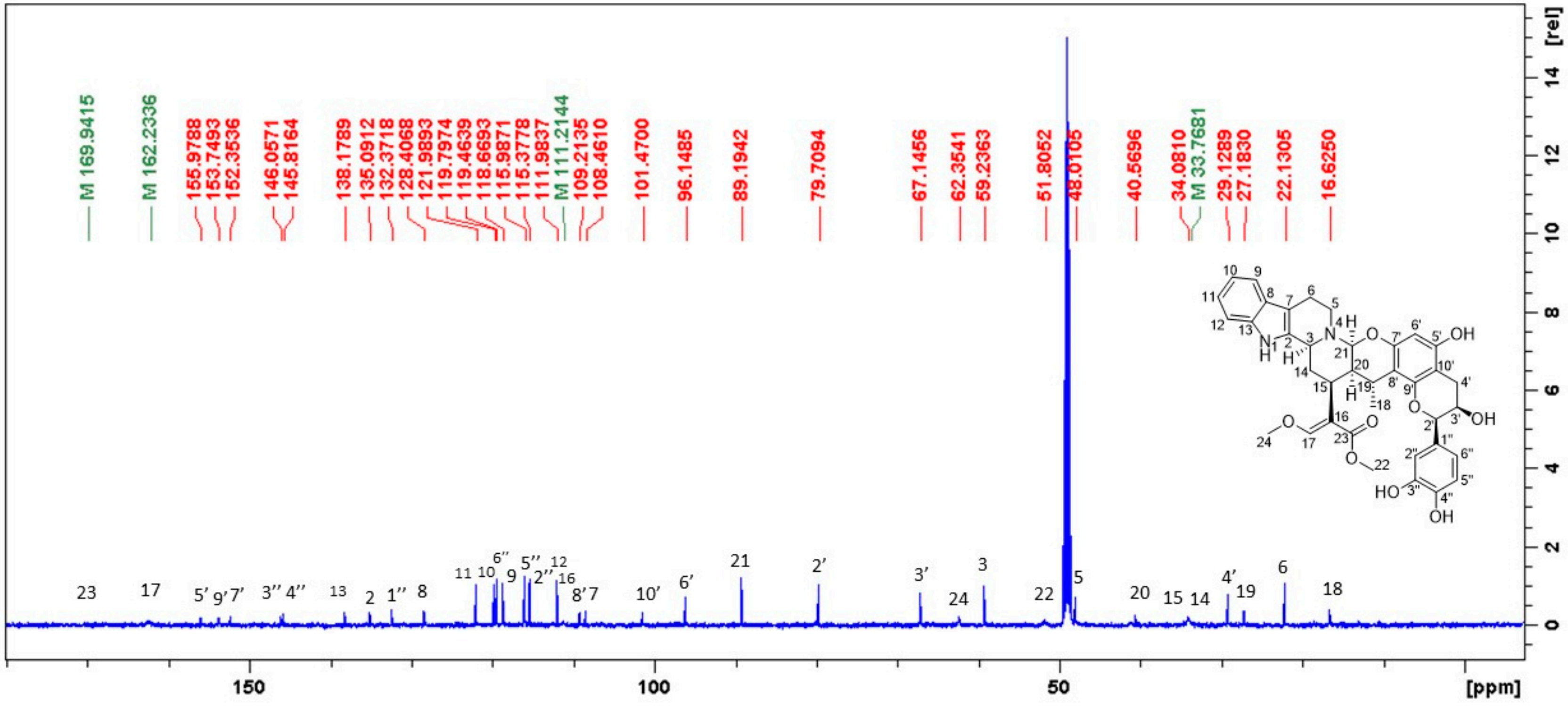

S30. COSY (CD3OD, 500 MHz) spectrum of **3**

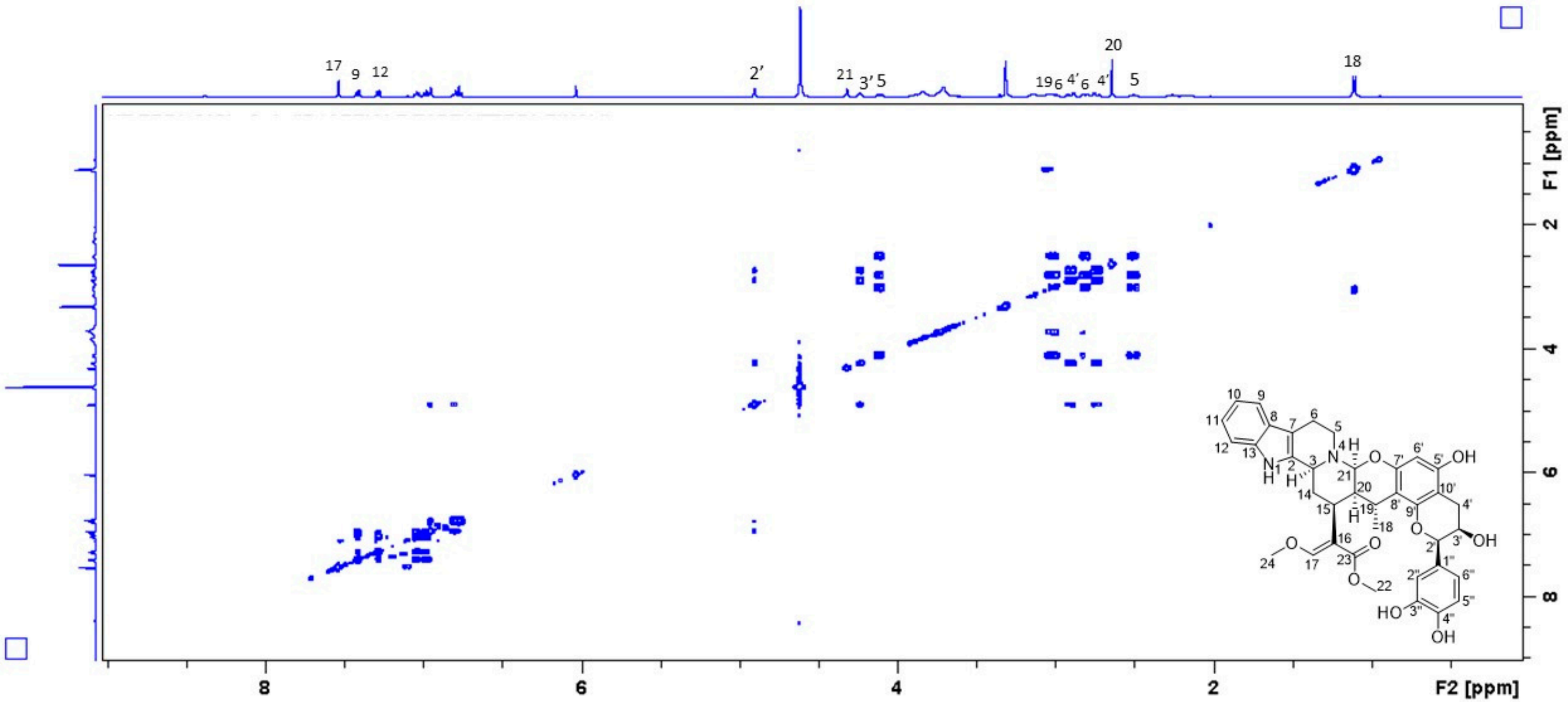

S31. COSY (CD3OD, 500 MHz) spectrum of **3** (1.6 to 3.9 ppm in ^{1}H dimension)

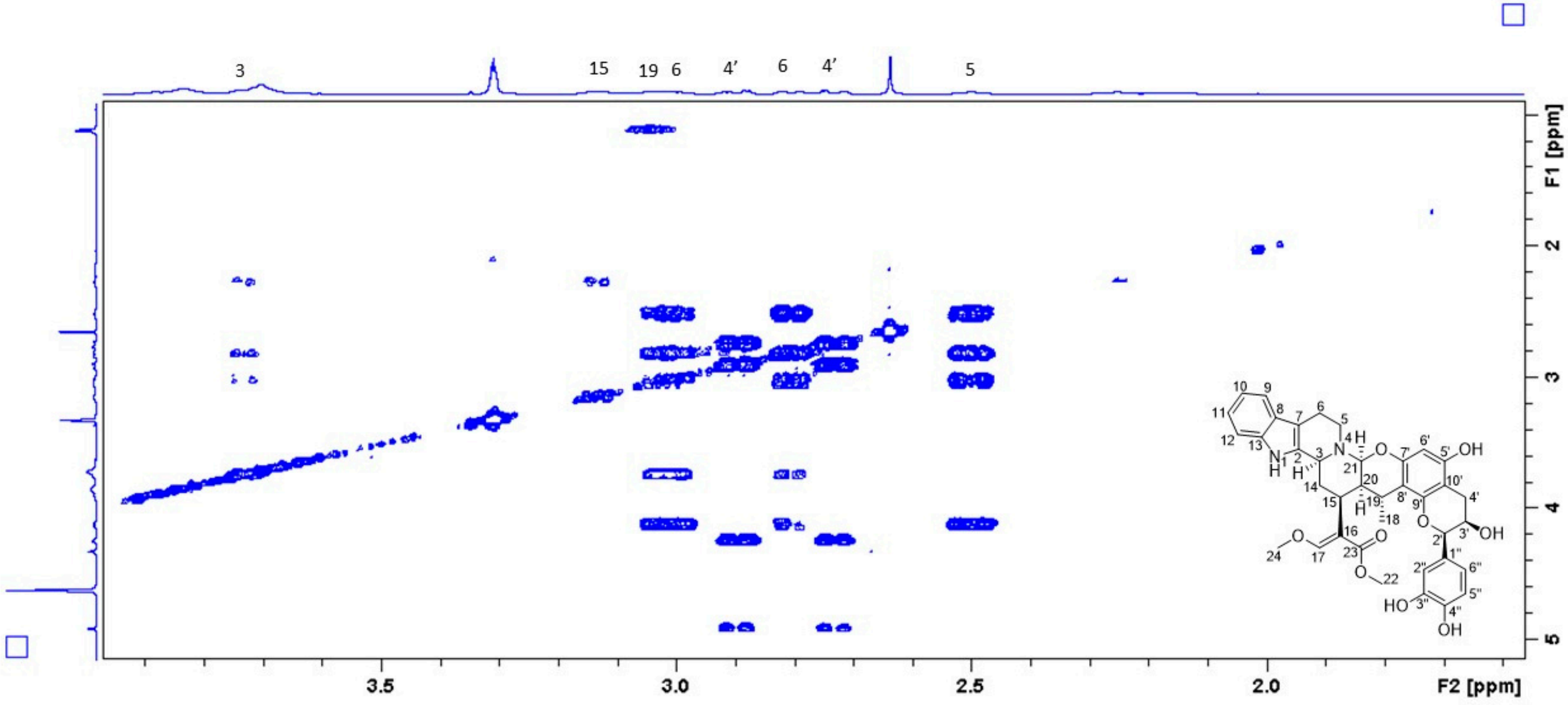

S32. HSQC (CD_3OD, 500 MHz, 125 MHz) spectrum of **3**

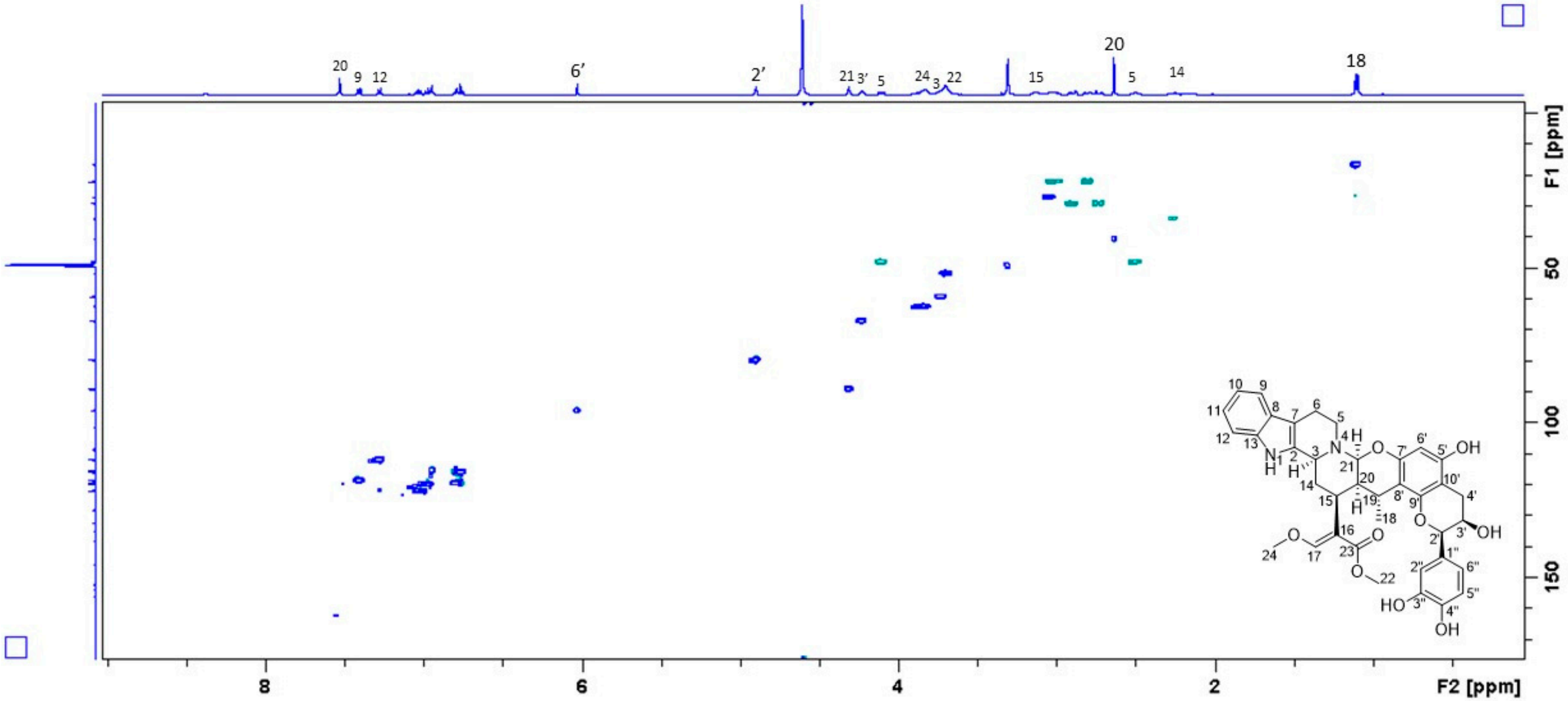

S33. HMBC (CD_3OD, 500 MHz, 125 MHz) spectrum of **3**

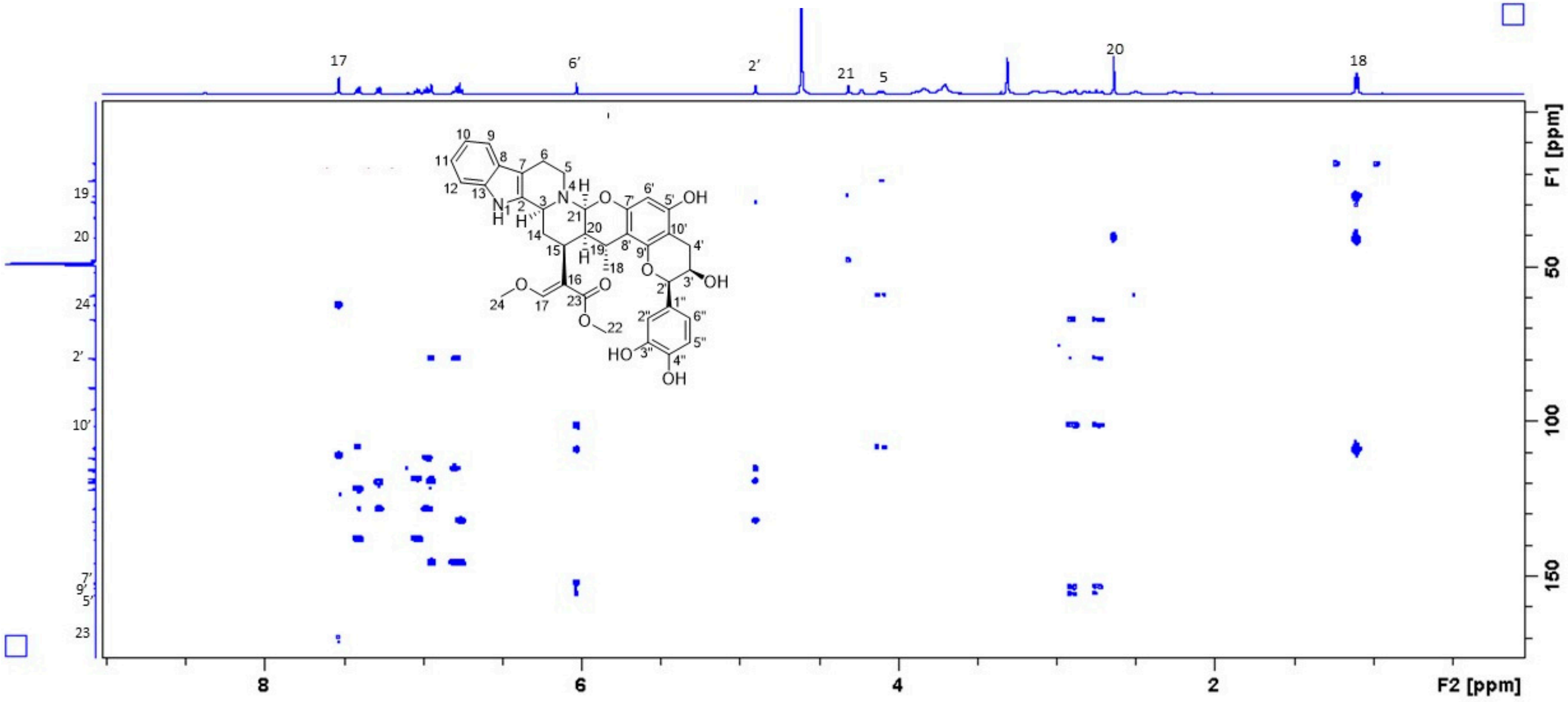

S34. HMBC (CD_3OD, 500 MHz, 125 MHz) spectrum of **3** (2.6 to 6.2 ppm in ^{1}H dimension)

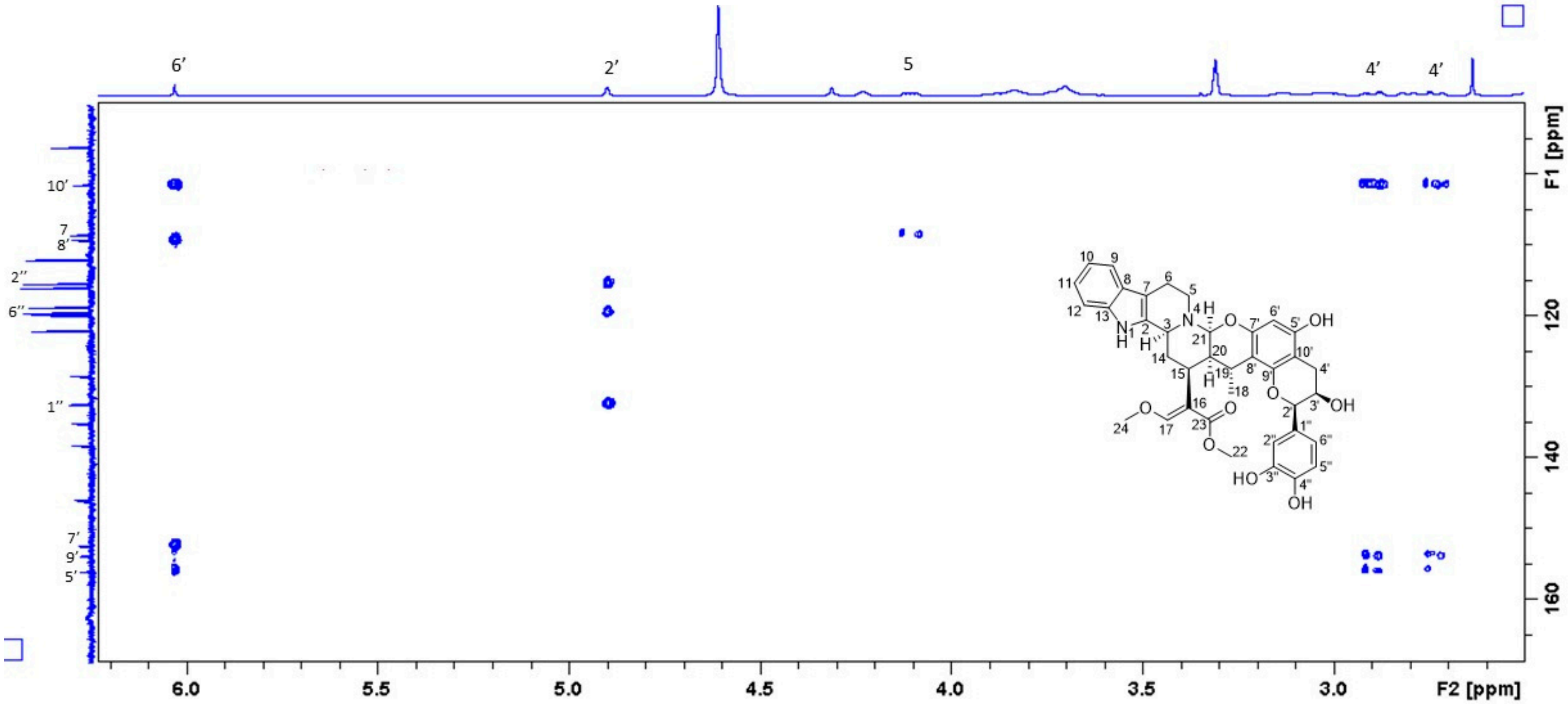

S35. ROESY (CD_3OD, 500 MHz) spectrum of **3**

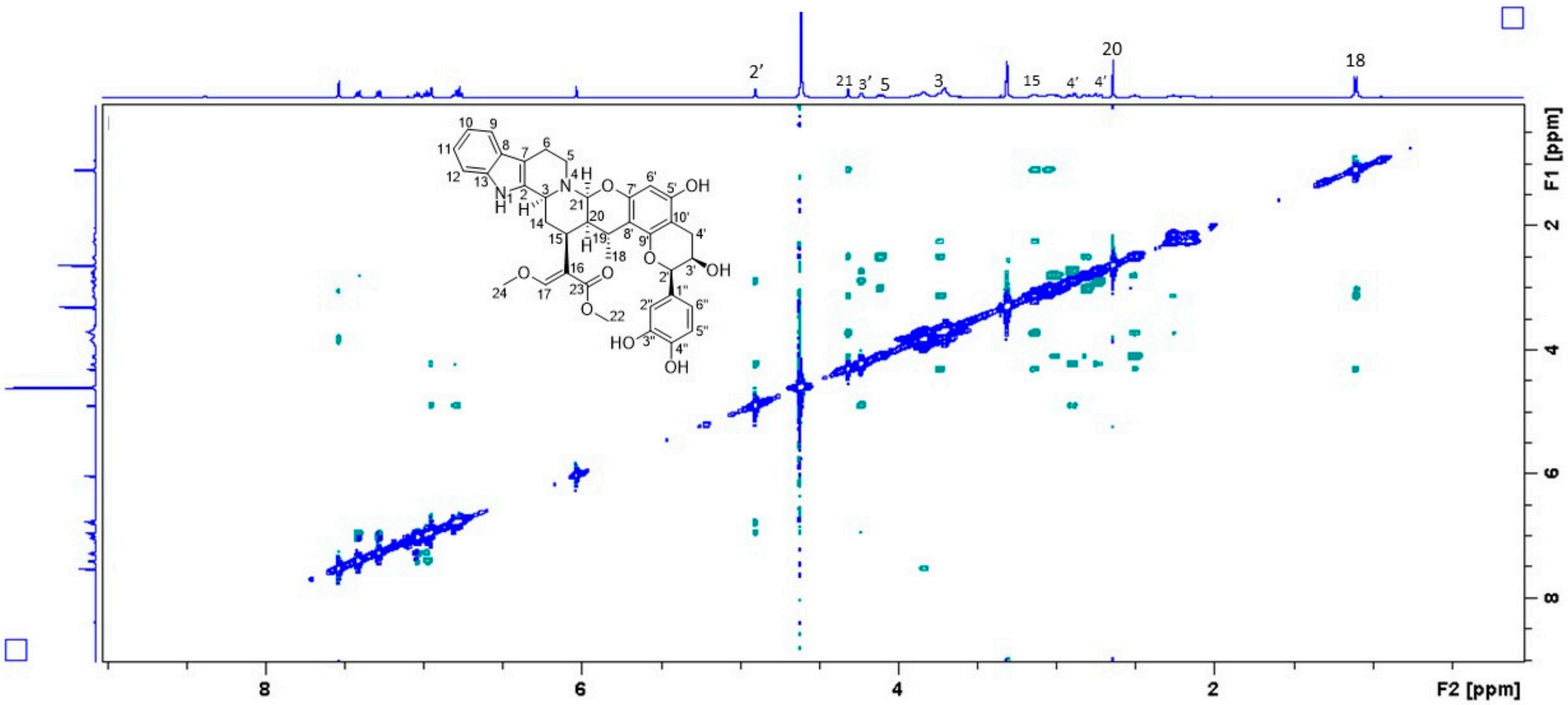

S36. ROESY (CD_3OD, 500 MHz) spectrum of **3** (expanded view)

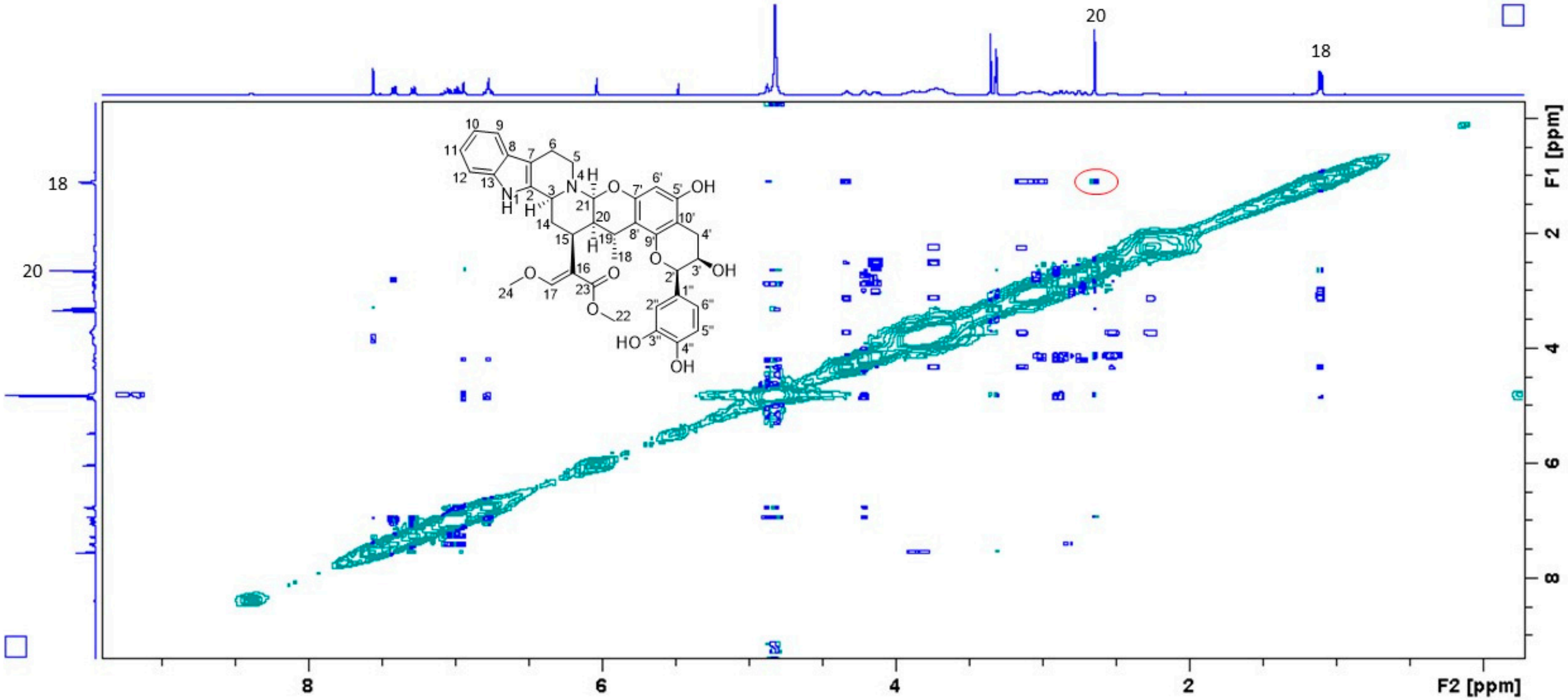

S37. Key ROE correlations of **3** (A: monoterpene indole alkaloid part; B: epicatechin part).

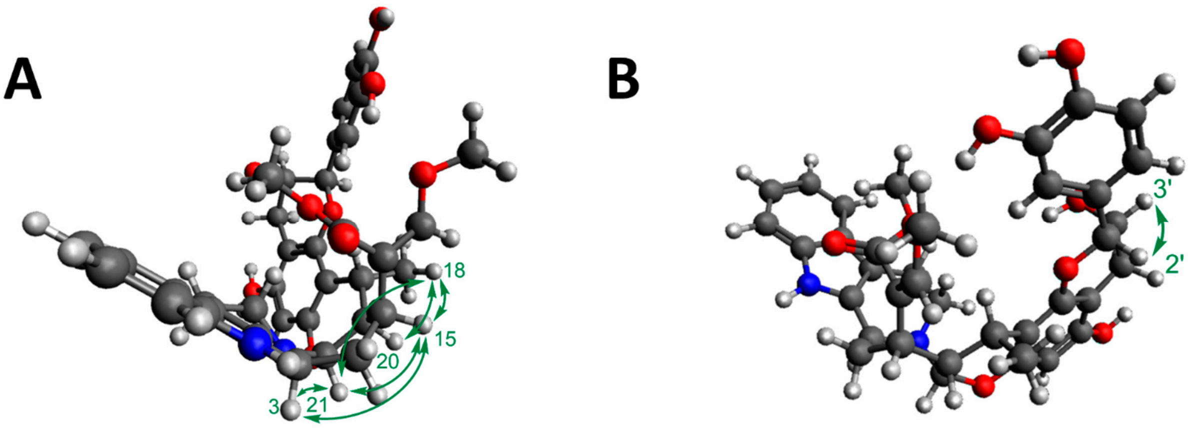

S38. Possible planar structures of epicatechocorynantheidine instigating two distinct linkages between the indolomonoterpenic alkaloid component and epicatechin

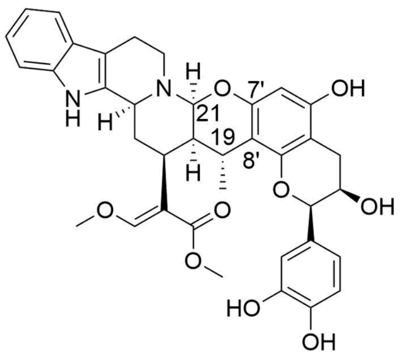

Epicatechocorynantheidine

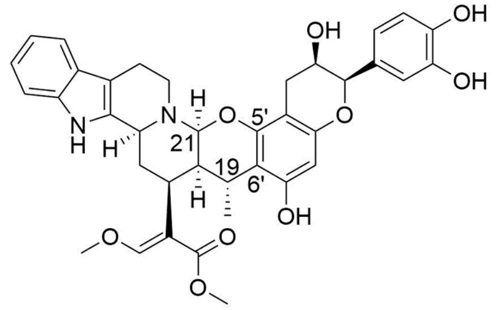

Alternative planar structure A

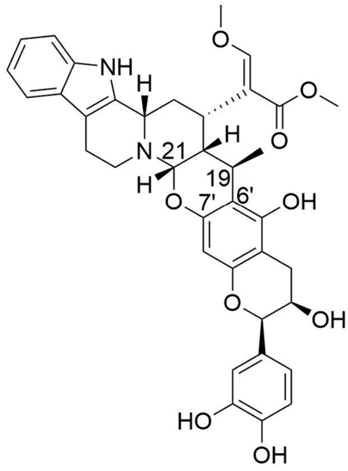

Alternative planar structure B

S39. Proposed biosynthetic scenario to **1**, **2** and **3**

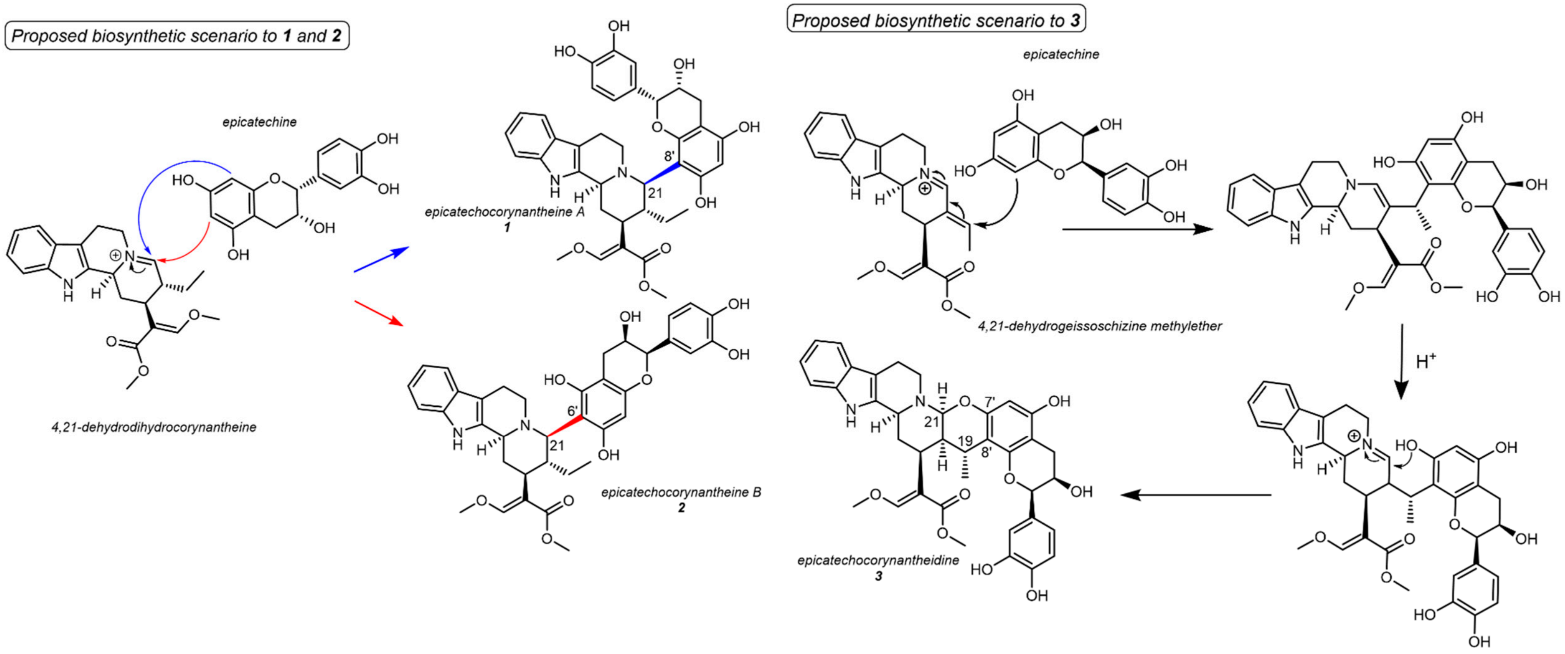

S40. HMBC correlation data of compounds **1-3**

H-position	**1**	**2**	**3**
3			
5			C-3, C-6, C-7
6			
9	C-7, C-11	C-7, C-11, C-13	C-7, C-11, C-13
10	C-8, C-12	C-8, C-12	C-8, C-12
11	C-9, C-13	C-9, C-13	C-9, C-13
12	C-8, C-10	C-8, C-10	C-8, C-10
14			
15			
17	C-15, C-16, C-23, C-24	C-16, C-24	C-16, C-23, C-24
18	C-19, C-20	C-19, C-20	C-8′, C-19, C-20
19	C-20		
20			
21	C-8′, C-9′, C-15, C-20	C-6′	C-5, C-19, C-7′
22	C-23	C-23	
24	C-17		
2′	C-3′, C-4′, C-9′, C-1′′, C-2′′, C-6′′	C-4′, C-9′, C-1′′, C-2′′, C-6′′	C-1′′, C-2′′, 6′′, C-4′, C-9′
3′	C-10′	C-10′	C-10′
4′	C-2′, C-3′, C-9′, C-10′	C-2′, C-3′, C-5′, C-10′	C-2′, C-3′, C-5′, C-9′, C-10′
6′		-	C-5′, C-7′, C-8′, C-10′
8′	-	C-6′, C-7′, C-9′	-
2′′	C-2′, C-1′′, C-4′′	C-2′, C-4′′, C-6′′	C-2′, C-4′′, C-6′′
5′′	C-1′′, C-3′′	C-1′′, C-3′′	C-1′′, C-3′′
6′′	C-2′, C-2′′, C-4′′	C-2′, C-2′′	C-2′, C-2′′, C-4′′

S41. GNPS Library Spectrum Accession Codes of the Isolated Compounds

Compound	GNPS Accession Code
Epicatechocorynantheine A	CCMSLIB00005721087
Epicatechocorynantheine B	CCMSLIB00005721088
Epicatechocorynantheidine	CCMSLIB00005721089
